# Understanding Inclusion and Participation of People From Black African Diaspora Communities in Health and Care Research: A Realist Review

**DOI:** 10.1111/hex.70298

**Published:** 2025-05-22

**Authors:** Eleanor Hoverd, Sophie Staniszewska, Jeremy Dale, Dawn Edge, Rachel Spencer, Violet Effiom, Dionne Gravesande, Lorna Hollowood, Samantha Johnson, Tony Kelly, Roy McFarlane, Esther Mukuka, Shane Ward

**Affiliations:** ^1^ Warwick Applied Health, Warwick Medical School University of Warwick Coventry UK; ^2^ Department of Health and Social Studies, Royal College of Nursing Research Institute University of Warwick Coventry UK; ^3^ Division of Psychology and Mental Health University of Manchester Manchester UK; ^4^ West Midlands Regional Research Delivery Network Wolverhampton UK; ^5^ Public Contributor Northampton UK; ^6^ School of Nursing and Midwifery University of Birmingham Birmingham UK; ^7^ Public Contributor Birmingham UK; ^8^ Public Contributor Brighton UK; ^9^ National Institute for Health and Care Research Leeds UK; ^10^ Public Contributor Sandwell UK

**Keywords:** Black people, co‐production, health and care research, inclusion, participation

## Abstract

**Background:**

People from Black African Diaspora Communities (BAFDC) experience poorer health outcomes and are persistently under‐represented in health and care research. There is limited understanding about how to support their greater inclusion and participation.

**Objectives:**

Explore secondary data providing insights for the co‐development of a realist theory of inclusion and participation for people from BAFDC in health and care research in the United Kingdom. Drawing on these theories, co‐produce a realist review with a diverse range of people from BAFDC.

**Methods:**

A realist approach underpinned the study. Pawson's five steps to a realist approach were taken to shape the review, identify relevant sources, extract the data and then analyse and synthesise to inform an overarching programme theory. Initial programme theories (IPTs) were developed through context (C), mechanism (M), outcome (O) configurations (CMOCs).

**Main Results:**

The review identified 43 relevant documents. Synthesis of evidence from the documents resulted in 8 IPTs and 17 CMOCs helping to understand and explain the inclusion and participation of people from BAFDC. Four key thematic clusters emerged: (1) *Health and care research as a White space,* (2) *Trust deficit: the expansiveness of broken trust,* (3) *Implicit and complicit bias* and (4) *Processes that affect inclusion and participation*. Findings were underpinned by five existing mid‐range theories (MRTs) around central concepts of candidacy, social dominance, networks, narratives and racism that guided analysis and synthesis, supporting conceptualisation of CMOCs. An overarching programme theory was developed.

**Conclusion:**

The review identifies how the influence of perspectives, attitudes and beliefs held by individuals or groups about people from BAFDC operates in health and care research, resulting in exclusion, lack of trust and deficit thinking. The findings should be used to inform interventions aimed at increasing inclusion and participation of people from BAFDC.

**Patient or Public Contribution:**

The co‐production group comprised a diverse range of individuals from within the health and care research system with different lived experiences of being Black. They contributed to the entire review process, including the development of initial programme theories and retroductive thinking and interpretation of the evidence.

**Clinical Trial Registration:**

Not applicable.

## 1 Introduction

In the United Kingdom, it is recognised that the inclusion and participation in health and care research of people from Black African Diaspora Communities (BAFDC) is of paramount importance as they encounter some of the greatest health inequalities, which may have serious consequences on their health outcomes [1, 2, 3, 4, 5, 6, 7, 8, 9]. BAFDC are a minoritised group that includes Black, Black British, Black Welsh, Caribbean or African individuals with dual/multiple heritage and other groups who have Black and African lineage [1]. It is important to recognise that there are significant social and cultural differences within BAFDC, which is necessary in understanding the different needs of individuals within BAFDC [10, 11]. To understand why disparities in health exist in this population, it is essential that people from BAFDC are included in health and care research [12, 13].

The attention towards research equity and ‘whom’ is taking part in health and care research has become a key priority and challenge for many health and care research systems around the globe [14, 15, 16, 17, 18, 19]. However, it is still not well understood why greater inclusion and participation of people from BAFDC in health and care research have not been achieved [1, 3, 14, 16, 17, 18]. While recognising that definitions of equality, diversity and inclusion (EDI) vary internationally, for the purposes of this study, we drew on those of the National Institute for Health and Care Research (NIHR), the largest funding body of research in the United Kingdom [20]. These are summarised in Table 1.

**Table 1:** Definitions of important terms.

Term	Definition
Equity	Aiming to understand and give people what they require to achieve their potential. This includes promoting notions of fairness, justice, entitlements and rights [20].
Equality	Ensuring everyone is given equal access to resources. This also includes ensuring equal opportunities are given to allow people to utilise their skills and talents [20].
Diversity	Being reflective of the wider community through a diverse community, which involves people from a broad range of backgrounds represented in all areas and at all levels [20].
Inclusion	An approach where groups or individuals from different backgrounds are welcomed, culturally and socially accepted, and treated equally. This means engaging with each person as an individual and creating a sense of belonging that is respectful of people for who they are [20].
Participation	When ‘*people take part in a research study*’ either testing new treatments or devices, or completing surveys [21].
Involvement	When patients and members of the public work together as partners with researchers to shape a research project [21].

In England, the NIHR has made it a key priority implementing its Research Inclusion Strategy 2022–2027 and funding the INCLUDE (Innovations in Clinical Trial Design and Delivery for the Underserved) project to better understand the barriers to participation by underserved groups who have lower inclusion in health and care research studies, developing guidance to support access to health and care research [12, 13, 15, 20, 22].

A realist review seeks to explain what works, for whom, how, why and under what circumstances [23]. The overall aim of this realist review was to broadly explore a range of secondary literature sources to contribute to the development of programme theories and then work with a co‐production group to refine and test them, as well as explain the context of the health and care research system in relation to inclusion and participation of people from BAFDC [1, 23]. This review is part of a wider realist evaluation to answer the research questions published in the protocol, which will ultimately support the development of a specific intervention or programme to facilitate the inclusion and participation of people from BAFDC in health and care research. Research questions are displayed in Box 1 [1]:

Box 1 **Realist review questions [1].**

1. What are the contextual and/or causal factors that influence inclusion and participation in health and care research by people from BAFDC?
2. How and where do mechanisms occur that underlie implicit and complicit bias that affect the inclusion and participation of people from BAFDC in health and care research?
3. What barriers and facilitators do people from BAFDC feel affect their experiences of participation in health and care research?
4. What might the components of an intervention(s) look like that foster inclusion and participation of people from BAFDC in health and care research?

## 2 Patient and Public Involvement

A GRIPP2‐SF can be found in Supporting Information File 1, reporting how members of the public were involved as research partners at all stages of the realist review. A co‐production approach was taken, involving eight individuals from around the health and care research system (V.E., D.G., L.H., T.K., E.M., R.M., I.S. and S.W.) with different lived experiences of being Black. Some of these individuals were public contributors. E.H. facilitated and attended all co‐production group meetings.

The co‐production group was consulted throughout, and 10 virtual meetings were held on Microsoft Teams, as well as 1 face‐to‐face meeting. Four more ad hoc meetings were held for members of the group who were not able to attend on particular dates. Virtual meetings lasted 1‐2 h with the face‐to‐face meeting lasting 4 h to allow time for building relationships as well as spend more time on lengthier discussions. The meetings aimed to ensure everyone felt valued, like they belonged, understood co‐production and were included. Public contributors were reimbursed according to NIHR payment guidance for researchers [24]. Co‐production activities are shown in Box 2.

Box 2 **Co‐production activities.**

- Initial introduction to each other
- Agreeing on preferred methods of communication, place, structure, schedule of meetings, and roles and responsibilities
- NIHR co‐production training
- Lego Serious Play for rough initial programme theory (IPT) development
- Shortlisting existing mid‐range theories (MRTs)
- Development of the realist review protocol and manuscript
- Co‐production of an ethical framework
- Discussion of findings with a plan for further searching and development of IPTs

## 3 Methods

A detailed realist review protocol was registered in PROSPERO (registration no: CRD42024517124) and published in combination with the Realist And MEta‐narrative evidence Synthes: Evolving Standards (RAMESES) for realist reviews [1, 25, 26]. Ethical approval was not required. A brief summary of the review protocol is provided in this section. The realist review followed Pawson's [25] five recommended steps, with each step described below.

### 3.1 Step 1: Shaping the Scope of the Review

The goal of Step 1 was to shape the review with the co‐production group and research team by identifying key concepts of relevance through a process of ‘concept mining’ using Lego Serious Play (LSP) [27]. LSP encouraged the co‐production group to reach a deeper level of thinking about how research participation is currently working for people from BAFDC [27, 28, 29]. Through building Lego models, co‐production members produced tangible, metaphorical figures representing the research participation pathway, creating focus points for discussion [1]. The group described the research participation pathway as ‘*inaccessible, complex, uncertain, tokenistic, exclusive and divided*’ and one that is viewed as a pipeline for White people only. Through discussion, key concepts were identified that were felt to be important to people from BAFDC in relation to how inclusion and participation could work in health and care research, informing the development of a rough initial programme theory (rIPT) shown in Box 3 [30].

Box 3 **Rough initial programme theory (rIPT).**

***If*** health and care research does not prioritise topics or areas of relevance that affect Black African Diaspora Communities in the United Kingdom (due to their lack of involvement in prioritisation) who have faced racism, racial discrimination, been historically neglected, historically ignored, marginalised and excluded ***then*** people from BAFDC do not take part ***because*** research studies offered are not related to their experiences, or their needs leading to under‐representation in health and social care research.

A mind map summarised the discussions with central concepts displayed in Table 2.

**Table 2:** Central concepts from LSP.

Concept	Explanation
Narratives	Narratives in relation to historical mistreatment and abuse of people from BAFDC, negative experiences of care in the NHS and campaigns that are developed with White people in mind.
Human experience	Human experience in relation to the negative experiences of healthcare and health and care research.
Service	Service in relation to navigating the healthcare and the health and care research system, and who they are designed for.
Oppression	Oppression, in relation to the denial that structural racism exists and too many barriers to overcome, resulting in exhaustion to get to the point of care/research opportunity.

Extensive dialogue from the LSP discussions led to agreement that these key concepts are of significant importance and informed a theoretical framework by searching for existing theories that would help to define these concepts further and those that the co‐production group felt were most relevant. E.H. examined the literature and conferred with experts from the realist field and international experts on inclusion and participation of people from diverse racial‐ethnic backgrounds. A list of existing MRTs was compiled by E.H. The co‐production group shortlisted these based upon the theories they considered to be most important (reason for consideration) and most relevant to the review questions (reason for inclusion). A process of shortlisting existing MRTs involved a presentation of each MRT to the co‐production group (by E.H.), followed by discussion and group voting. The reason for inclusion was determined by the co‐production group. Table 3 shows the resultant theoretical framework.

**Table 3:** Resultant theoretical framework.

Existing mid‐range theory (MRT)	Reason for consideration	Reason for inclusion
Candidacy theory	Candidacy concerns ‘*how people assess their eligibility for accessing health services and how they legitimise their interaction and engagement with services’* [31]	Felt to be an important theory for understanding how a person from a BAFDC comes to determine if they may be a candidate for research, as well as helping to identify key mechanisms required for a positive outcome (participation) [31]
Network theory	‘*People's behaviour is best predicted by examining not their drives, attitudes, or demographic characteristics, but rather the web of relationships in which they are embedded. That web of relationships presents opportunities and imposes constraints on people's behaviour’* [32]	Black networks are a strong influence in the decision‐making process as a way of supporting their communities [32]
Narrative theory	‘*The idea that sharing a story can add credibility and authenticity to health messages. The interplay between a person's self‐concept and the situation, containing the social forces emanating from other people and institutions that direct him/her how to think, feel, and behave is at the heart of the process of identification.’* [33]	Narratives may be a powerful context or mechanism that can influence individuals' identities or perception of themself. They can also influence the perceptions of people who do not have the lived experience of being Black, such as creating stereotypes or assumptions, and this impacts the health outcomes of people from BAFDC [33]
Social dominance theory	Social dominance centres on the way in which hierarchies are created, underpinned by beliefs and attitudes which reinforce ‘*institutional dominance*’ [34]	Based on the idea that the majority of societies comprise of hierarchies in which some people are more privileged than others, this theory may help to understand the causal factors that contribute to hierarchical relationships [34].
Critical race theory	Critical race theory ‘*examines the role of race, racism and power. Its roots are in legal studies and radical feminism—it is committed to eliminating all forms of oppression through an interdisciplinary approach.’* [35]	It may help understand the psychosocial mechanisms behind racial inequality in research [35, 36]

The theoretical framework was used to develop IPTs and the data extraction form (adapted from Rycroft‐Malone et al. 2012 and *CARES Realist Methodology Training* by Jagosh 2022 [28, 36]) aiming to support identification of context (C), mechanisms (M) and outcomes (O) in selected documents (Supporting Information File 2). IPTs formed a key aspect of analysis and overall programme theory development. As Pawson explains, a key aspect of undertaking realist research is to ‘*bring to the surface*’ a theory, or theories that help to explain what works, for whom, how and under what circumstances [25]. Programme theories were developed to explain the relationship between differing contexts (C) and mechanisms (M), which result in certain outcomes (O) [25]. Mechanisms are understood as resources within a programme or intervention that provoke a response by the user [26]. These hypotheses are represented as CMO configurations (CMOCs) that explain causality at a deeper level [25].

### 3.2 Step 2 Searching for the Evidence

**Figure 1:**
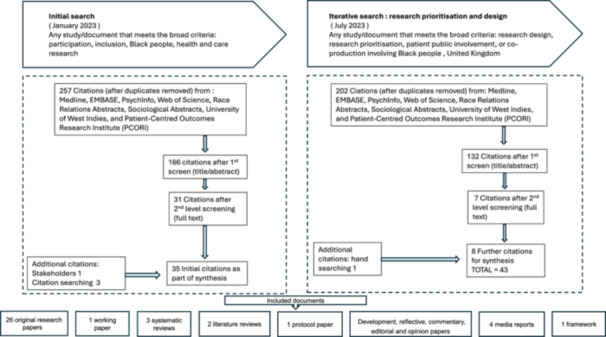
Flow diagram of initial search and iterative search.

Realist reviews support the opportunity to elicit evidence from a diverse range of sources [23]. The goal of realist searches is to capture relevant data that contributes to the development of programme theory; they are not intended to be exhaustive [37]. To gain a global understanding, evidence was collected from secondary data sources and grey literature from a range of countries (see Supporting Information File 3) [1, 38]. The initial search was carried out in Medline, EMBASE, PsycINFO, Web of Science, Race Relations Abstracts, Sociological Abstracts, University of the West Indies (UWI Scholar) and Patient‐Centred Outcomes Research Institute (PCORI) with search terms adapted for each database. Forward and backward citation tracking was conducted. Documents were not excluded on the basis of the date of publication, with searches including documents published from as early as 1946, if relevant, to capture evidence that would signify crucial historical and political contexts that may have influenced inclusion [1]. Evidence from the grey literature and key recommendations suggested by the co‐production group was also included. Realist reviews are iterative by nature [25, 29]. An iterative search was conducted in September 2023 following discussions of the emerging evidence with the co‐production group [28, 39, 40]. This focussed on research prioritisation, research design and patient public involvement (PPI) in the United Kingdom and was carried out using the same databases and limits (excluding University of West Indies Scholar and PCORI as scoping searches on these databases produced no relevant papers). Documents were included based on relevance to furthering understanding of causation and programme theory development [36, 38]. A complete list of the search strategies (initial search and iterative search is reported in Supporting Information File 4 [41]. A flow diagram in Figure 1 illustrates the search process.

Inclusion and exclusion criteria for the initial search, as reported in the protocol paper, and iterative search, are shown together in Table 4 [1].

**Table 4:** Inclusion and exclusion criteria for the initial search and the iterative search.

Initial search	Iterative search
Inclusion	Inclusion
No initial date parameter.	All dates included post‐1946.
All study designs (qualitative, mixed methods, quantitative, systematic reviews, etc.) and grey literature.	All study designs (qualitative, mixed methods, quantitative, systematic reviews, etc.) and grey literature.
All international sources will be included.	UK sources only included.
Document has relevance to the development of Programme Theory, either the full text or a section.	Document has relevance to the development of Programme Theory, either the full text or a section.
Population must be related to Black African Diaspora Communities (BAFDC), Black British, African American, Black African or Black Caribbean people, though this may include sources that include information about other ethnic minority groups as well.	Population must be related to Black African Diaspora Communities (BAFDC), Black British, Black African or Black Caribbean people, though this may include sources that include information about other ethnic minority groups as well.
Must concern factors that affect inclusion and participation in health and care research with health and social care research.	Must concern factors in relation to the development of research questions, and/or Patient Public Involvement and Engagement (including co‐production).
Exclusion	Exclusion
Relates to people from BAFDC, Black British, African American, Black African and Black Caribbean people	Relates to people from BAFDC, Black British, Black African and Black Caribbean people

The terms used to describe the race and ethnicity of people from BAFDC may not be exhaustive. These terms were based upon those most frequently cited in the sources, though it is understood that language regarding race and ethnicity is changeable and may risk conflating different groups of individuals [10, 11]. As the review sought to have international relevance, there was recognition that terminology also varies in other countries.

### 3.3 Step 3. Document Selection and Appraisal

Figure 1 shows a detailed flow diagram of the initial search and iterative search. References were exported to referencing software *Rayaan* to screen for duplicates [42]. E.H. screened documents by title and abstract, and the second reviewer, V.E., screened a random 10%. Full‐text screening of the 166 documents remaining was carried out by E.H. The data extraction form supported second‐level screening of full texts with documents selected relative to their relevance and rigour [1]. V.E. screened a further random selection of 10% of the full texts. A total of 43 documents were included following the initial search and iterative search screening [43, 44, 45, 46, 47, 48, 49, 50, 51, 52, 53, 54, 55, 56, 57, 58, 59, 60, 61, 62, 63, 64, 65, 66, 67, 68, 69, 70, 71, 72, 73, 74, 75, 76, 77, 78, 79, 80, 81, 82, 83, 84, 85]. Documents covered were representative of only the United Kingdom (40%) and the United States (60%), though one paper focussed on sharing community partnership initiatives in the United Kingdom and South Africa [83]. Publication dates ranged from 2004 to 2023.

### 3.4 Step 4 Data Extraction

E.H. read and re‐read the documents, discussing key aspects with the co‐production group and research team, studying the trustworthiness and rigour of documents included. Document characteristics (reference, year, country, type of document, aim, methods and participants) were summarised and are shown in Supporting Information File 5.

### 3.5 Step 5 Data Synthesis

**Figure 2:**
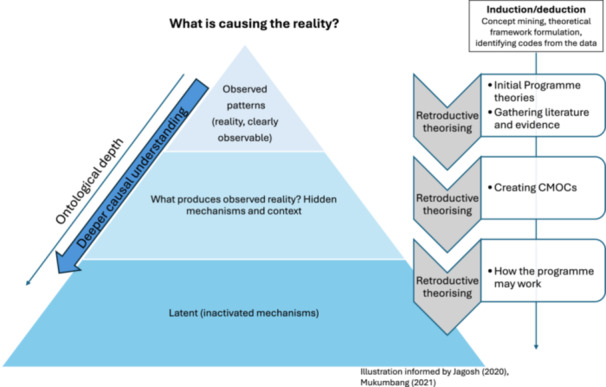
Retroductive theorising.

Analysis and synthesis of the data were undertaken by E.H. in discussion with the co‐production group and research team. Employing abductive and retroductive thinking to develop programme theory was essential in applying inductive and deductive logic, as per realist thinking [86, 87, 88, 89]. Retroduction supports theorising, through abstract thinking, helping to develop causal explanation, as opposed to searching for themes [87]. Figure 2 illustrates retroductive theorising based upon evidence from Jagosh (2020) and Mukumbang et al. (2021) [87, 88].

The data extraction forms, underpinned by the theoretical framework, supported a deductive process of thinking. Inductive analysis was undertaken through E.H. coding relevant sections of text with V.E., using NVivo data management software and manually annotating documents to identify contexts (C), mechanisms (M) and outcomes (O) [89, 90]. The theoretical framework was used as a basis to group the codes according to the existing MRTs.

An eclectic approach to analysing data through sketching diagrams, collating key quotes that were felt to capture important evidence supporting causal explanations, manually annotating papers and coding in NVivo was used to support analysis [38, 89, 90]. Box 4 shows an example of a key quote and how a mechanism was identified within it.

Box 4 **Example of key quote for identifying and analysing CMOCs.**

- ‘*Another participant talked about his perception of clinical trials and the perceived lack of caring from the researcher about the larger cause indicating, “A clinical trial, I don't think is safe…. Because they're researching, trying to get something, numbers. I feel like they're trying to get number[s] instead of actually getting a cause taken care of”.’* Griffith et al. 2020 [62].
- *Mechanism:* the relationship between the researcher and the potential participant (*resource*) was not perceived as caring and more focussed on recruitment, which made the potential participant feel unsafe (*response/reasoning*).

CMOCs were developed and organised into groups. IPTs were then co‐produced. Discussions around IPTs with the co‐production group and research team supported refinement of the IPTs through fostering abductive thinking or ‘hunch‐driven theorising’ that is described by Jagosh as an important aspect of realist thinking [36, 87].

Data synthesis was strengthened by juxtaposing sources to reveal further insights, merging sources where data indicated that mechanisms and outcomes appear to relate and judging sources based upon their methodological strengths and weaknesses [23]. Through synthesising and refining CMOCs and IPTs through drawing on the existing MRTs in the co‐produced theoretical framework, patterns of outcomes were identified in relation to the mechanisms and contexts and used to build an overarching programme theory. The findings of the realist review are described in the next section.

## 4 Results

**Figure 3:**
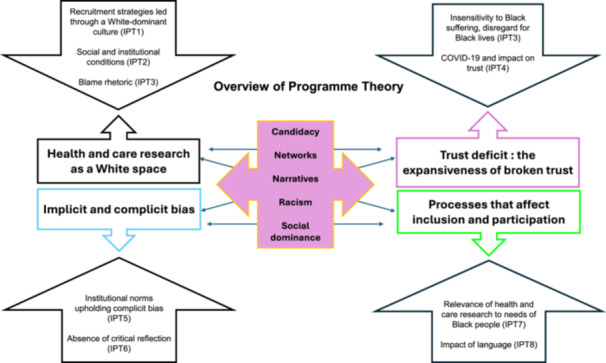
Programme theory.

From the 43 documents included, 8 IPTs were identified and 17 CMOCs, together informing an overarching programme theory (Figure 3) about inclusion and participation of people from BAFDC in the health and care research system. The process of forming these IPTs from the literature helps to understand the architecture of the health and care research system in relation to the inclusion and participation of people from BAFDC. CMOCs create the foundation for each IPT. The programme theory outlines the underlying causal assumptions that explain the contexts and the relationships with key mechanisms and the outcomes that result in the inclusion or exclusion of people from BAFDC in health and care research.

Four key thematic clusters emerged from the included evidence: (1) *Health and care research as a White space*, (2) *Trust deficit: the expansiveness of broken trust*, (3) *Implicit and complicit bias* and (4) *Processes that affect inclusion and participation*. Supporting Information File 6 details a table that begins with a key thematic cluster linked to the specific IPTs and CMOCs underneath, which support it [25].

The following section summarises the four main clusters, underpinned by existing MRTs, in relation to the programme theory developed from the realist analysis.

### 4.1 Health and Care Research as a White Space

In this first cluster, analysis indicates the inter‐relationships that exist between narratives (both spoken and visual) and networks within the health and care research system contribute to forming some of the hidden structures of a White space [34, 35].

Numerous documents included descriptions of the health and care research system as being a White space in *the way* in which it is designed and *who* it is designed for [4, 53, 54, 56, 60, 62, 65, 71, 72]. Groups who have disproportionately better health outcomes and reap the benefits of research tend to be White populations [46, 66, 91]. Leadership in health and care research is hypothesised to be predominantly led through a White culture, privileging White populations, which can be further understood through social dominance theory [34].

Social dominance theory purports that an arbitrary‐set system, which is culturally defined, includes race and ethnicity as a socially constructed category [34]. This results in some groups having more power and privilege than others, with key mechanisms of individual and institutional discrimination and behavioural asymmetry maintaining oppression [34]. These are context‐specific depending upon settings and the values, attitudes and beliefs that organisations or individuals view as the norm. Social dominance also has a significant influence on an individual's candidacy. The mechanism of behavioural asymmetry, for example, relates to how individuals self‐categorise themselves and can influence one's candidacy for a service [34]. Social dominance also influences the ability to obtain funding.

Interracial hierarchies may be sustained within some institutions and organisations, resulting in White individuals benefiting from more funding [65, 69, 83]. A causal factor for this may be hegemonic Whiteness or the positionality of some White people viewing themselves as more important, whilst marginalising groups who are not White [92, 93, 94]. However, hegemonic Whiteness may be context‐specific and vary according to location and points in time, for example, how mature an organisation may be in relation to anti‐racism or EDI [76, 92, 95].

When Whiteness becomes centralised within the system, it impacts the social and institutional conditions affecting *who* takes part, with evidence suggesting that racialised hegemony (a form of racial domination) is a mechanism that excludes people from BAFDC [58, 60, 75, 84]. Racialised hegemony stifles inclusion because of the impact of social dominance through a lack of ethno‐racial diversity in the scientific workforce and a lack of response to the needs of people from BAFDC, leading to policies developed based on the needs of White people [58, 60, 69, 75, 76, 84]. This creates interracial tensions because of a lack of improvement in the wider social determinants of health [58, 60, 69, 75, 84].

A lack of ethno‐racial diversity can also be seen amongst commissioning teams, researchers and participants in research, heavily influencing recruitment strategies in a way that does not meet the needs of people from BAFDC [94, 95, 96]. This may be related to recruitment targets perpetuating inequalities, creating pressure through prioritising speed and higher enrolment, leading to health and care researchers taking research to places where populations are deemed easier to recruit from, and feeding the ‘hard to reach’ narrative resulting in the exclusion of people from BAFDC [48, 51, 55, 56, 57, 58, 59, 60, 61, 62, 63, 64, 65, 69].

Harmful narratives such as ‘hard to reach’ indicate assumptions made about people from BAFDC, further eroding trust [48, 51, 55, 60, 62, 64, 69, 73]. This deficit thinking is closely related to the concept of Whiteness [48, 60, 69, 72, 73, 74, 80, 81, 82]. Whiteness supports a blame rhetoric as a result of stereotypes and racial bias, whilst justifying the marginalisation of people from BAFDC by placing the responsibility on people from BAFDC to trust health and care researchers [48, 60, 69, 72, 73, 74, 81, 82]. However, there is evidence to suggest that through counter‐storytelling, deficit narratives about people from BAFDC can be exposed and counteracted, building trust and supporting social justice [77].

### 4.2 Trust Deficit: The Expansiveness of Broken Trust

Without acknowledgement of the impact of racism on Black people's lives, combined with a lack of trusting relationships with researchers and research institutions, trust continues to be broken. Analysis in the second cluster suggests that the continuum of broken trust that people from BAFDC face is a pre‐existing context stemming from legacies of colonialism and slavery, historical medical abuse and experimentation, racism and ongoing cultural trauma preventing progress in inclusion and participation [47, 56, 58, 62, 66, 70, 75, 83]. Distrust can be triggered as a coping mechanism in response to racism and discrimination experienced, which may be a natural response to an invitation to take part in research [51, 52, 83]. Many people from BAFDC do not feel that their lives are valued and are not provided with opportunities to be listened to or be involved in health and care research and healthcare more broadly, resulting in narratives of distrust that uphold mistrust in health and care research [45, 46, 47, 51, 58, 62, 66, 69, 73, 75, 78, 83, 84, 85].

Realist analysis in this cluster explains that because racism is so deeply entrenched within UK culture, society struggles to ‘see’ racism and speak openly about it, because of ideologies such as colour‐blindness (an ideology that ignores the existence of racism) and complicit behaviour [45, 53, 55, 57, 58, 62, 70, 73, 84].

Operating conditions at the macro level of the health and care research system and wider healthcare system, in relation to candidacy, may influence access, belonging, trust and consequently decision‐making about taking part in health and care research by people from BAFDC [95]. Relationships that health and care researchers have, *or* do not have, with people from BAFDC may create a barrier to engaging with health and care research [52, 97]. Previous negative interactions with healthcare professionals can also create barriers to participation [45, 60]. The credibility of health and care researchers and research institutions is an important factor that influences trust, with ‘word of mouth’ a key mechanism for creating narratives of cause for concern, or confidence and trust [55, 56, 57, 58, 59, 60, 62, 64, 75, 83, 85]. Credibility and trustworthiness are also influenced by a lack of visibility of how research findings are implemented and actioned to improve health outcomes for people from BAFDC. This may lead to feelings of fatigue and a lack of participation [56, 70, 73, 83]. Analysis highlights the importance of researchers having cultural humility, being racially competent and investing in relationships with people from BAFDC with a view to developing research studies that meet their needs and make them feel valued and cared about [45, 56, 59, 63, 75, 78, 80, 83]. This can improve credibility and trustworthiness [62, 85]. The development of strong ties or relationships with individuals, whether they are community members or professionals from within a system, can have a significant influence on trust [45, 55, 56, 75]. At the macro level of a system, trust can be influenced by an individual or organisation's reputation, with familiarity being a key mechanism to building trustworthiness [55, 60, 98].

Not appreciating how relationships are formed with people from BAFDC and not valuing the development of these relationships through PPI or co‐production may inhibit the mobilisation of communication and creation of strong ties [71, 98]. This may be due to health and care researchers not having the experience or confidence of working with people from BAFDC, creating a significant barrier to the development of strong ties.

### 4.3 Implicit and Complicit Bias

Analysis of cluster three suggests that the institutional norms that exist and lack of critical reflection by health and care researchers may be contributing to complicit and implicit bias upheld through harmful narratives often influenced by ideologies that are steered by socially dominant groups [45, 58, 60, 64, 66, 68, 70, 73, 75, 77, 84, 98, 99, 100]. The continued under‐representation of people from BAFDC in health and care research is theorised to be a result of complicit bias upheld by organisational or institutional norms and driven by productive ignorance [61, 65, 71, 74, 81, 82]. Productive ignorance centres on getting the job done, for example, reaching recruitment targets, though this prevents opportunities for researchers to discuss practices that might be structurally racist, and in some cases, researchers may even be discouraged from exposing structural racism [60]. Organisational norms influence this complicit behaviour and include unspoken standards, rules or accepted behaviours formed by dominant groups and are difficult to disrupt due to a lack of acknowledgement and discussion about structural racism. Political narratives are suggested to steer views and ideologies about the existence of structural racism, with evidence indicating some political narratives support a post‐racial stance with racism only existing at the individual level [45, 46, 60, 73]. This silencing creates an imbalance of power and shifts blame onto Black people, with interventions targeted at improving recruitment aimed at the individual level. Many narratives act as mechanisms that influence the inclusion of people from BAFDC in health and care research.

Existing narratives of racism may occur in the way in which scientific information is communicated, for example in medical journals through homogenisation of data that reflects White data as the norm and may contribute to the formation of strong narratives about health inequalities and how they are understood by health and care researchers [12, 33, 34, 75]. There may also be a lack of understanding of race as a social construct, through scientific discourses (or a lack of) that can lead to such beliefs and the creation of implicit bias [18, 73, 80]. Implicitly held biases by some health and care researchers that stem from scientific racism may influence their beliefs that Black people are biologically different, leading to harmful narratives that may exclude people from BAFDC from taking part in research [60, 64, 67, 68, 70, 75, 77, 79, 84]. The historical formulation of these narratives has led to the development of clinical guidelines founded upon these views, resulting in clinical decisions based upon ethnic descriptors that may be worsening health inequalities. A report by the NHS Race and Health Observatory on the under‐representation of people from Black, Asian and ethnic minority populations in genomics research has brought this to the fore [101]. The belief that there are biological differences between people from different races is upheld through the mechanism of cultural hegemony, maintaining racial hierarchies [60, 75, 84]. A lack of time spent on building relationships with people from BAFDC, in combination with beliefs that biologise (attributing differences to biological factors) and pathologise Black people going unquestioned, can lead to feelings of being dehumanised and treated like guinea pigs [68, 84].

It is theorised that if health and care researchers were to improve their knowledge of cultural differences and reflect upon their own racial identity and privileges, this would support more inclusive ways of working [45, 58, 60, 64, 66]. Reasons for this lack of deep critical reflection may occur due to White fragility and the fear of using racialised language, as well as a colourblind ideology that prevents disruption of the status quo and results in complicit behaviour [45, 58, 62, 64, 66, 93, 100, 102, 103, 104]. White fragility is a key mechanism that prevents action‐focussed conversations about race and may be hindering progress, around inclusion and participation, requiring further understanding about suitable interventions to support White health and care researchers to address this behaviour [102, 103, 104, 105]. White fragility causes discomfort in talking about racism and fear of repercussions or being accused of racism [102, 103]. White fragility is defined as an emotional state whereby White people protect themselves from racial stress through ‘*living, learning and working in predominantly white spaces, or by refusing to engage with the realities of race*’ [102]. Whilst White people may have this protective space, people from BAFDC are not able to protect themselves from racial stress, and thus, this may lead to interracial tension and a lack of trusting relationships with some researchers, affecting participation.

When critical reflection is based upon a context that creates an emancipatory and social justice praxis, it is felt by White anti‐racists who have experienced resistance by other White people, to aid in disrupting the status quo through action and could be an important consideration for the development of an intervention [102, 104]. The final cluster shares evidence around the processes that affect the inclusion and participation of people from BAFDC.

### 4.4 Processes That Affect Inclusion and Participation

Cluster four analysis implies further deficits in the existing wider contexts. Research prioritisation, PPI, wider historical policies and practices, openness, and the use and impact of language were all aspects of processes that affect inclusion and participation. Processes used to prioritise research areas and develop research questions through PPI may influence inclusion and participation of people from BAFDC, with evidence reporting that PPI is not seen as scientific, and subsequently undervalued [80, 82]. Some researchers may not see building relationships with people from BAFDC as scientific, with some lacking interest in PPI and developing their interpersonal skills to support cultural humility which can lead to people from BAFDC feeling that researchers do not want to work with them resulting in a lack of trust, keeping cultural barriers in place between researchers and people from BAFDC [45, 56, 66, 71, 83, 84, 85]. The prioritisation of research areas for funding and the development of research questions should align with the needs of people from BAFDC, but requires effective PPI or co‐production that involves a sharing of power and responsibility.

Historical policies, practices and social exclusion have negatively impacted people from BAFDC in the United Kingdom, such as the Windrush Scandal [44, 45, 46, 47, 48, 49, 50, 51, 58, 64, 73, 75, 83, 84]. Political narratives that transpose through policies and perpetuate structural racism can lead to feelings of rejection, instability and a lack of belonging by people from BAFDC, resulting in a lack of trust [34, 44, 46]. If policies that perpetuate structural racism are part of strong political narratives, then these may prevent change from occurring due to a lack of acknowledgement that some policies are upholding structural racism [54, 55, 60, 63].

Critical race theory demonstrates that racism is deeply embedded within the policies, practices and structures of the health and care research system [35, 94, 95, 100]. To individuals who are not Black, structural racism may not appear to exist because it is so deeply entrenched within structures and policies [54, 94]. Therefore, creating opportunities to identify and discuss racism is crucial in making progress [62, 76].

Finally, it is theorised that the importance and impact of language, identifying that when people from BAFDC receive information about health and care research that does not reflect their needs, results in a lack of trust and a lack of feeling that the research is for them. This may be due to the misalignment of organisational norms influencing how information is communicated, with a lack of appreciation of cultural differences, relevance and competence [48, 51, 52, 56, 64, 66, 72, 83, 85]. This has a detrimental effect on how safe people from BAFDC deem the research to be [56, 62].

## 5 Discussion

The ongoing under‐representation of people from BAFDC in health and care research continues to contribute to disparities in health outcomes. This realist review developed a programme theory which explains the under‐representation of people from BAFDC in health and care research underpinned by a co‐produced theoretical framework to deepen our understanding for developing an inclusive health and care research system, directly addressing the NIHR's recent commitment to making inclusion a condition of funding [105]. Major contextual considerations were identified around *who* controls the health and care research system and *how* the inter‐relationships between power and privilege, narratives, networks and trust influence trust itself. The processes and policies related to health and care research and how implicit and complicit bias contribute to the exclusion of people from BAFDC are explained and brought together into a single overarching programme theory. CMOCs 1–17 explain key mechanisms and how they are intertwined in the relationships (or lack of) between health and care researchers, policymakers, funders and people from BAFDC and ultimately influence trust.

### 5.1 Strengths and Limitations

E.H. is White British and acknowledges the power and privilege that comes with her racial identity and has reflected on her positionality throughout the realist review. Therefore, de‐centring Whiteness and centring Black voices through taking a co‐production approach is a key strength of this realist review, with the group's lived experiences of being Black, marginalised, historically neglected, ignored and excluded making them experts in their own right [1, 106]. A culture of openness at co‐production meetings enabled E.H. to discuss her own assumptions and biases, acknowledging any mistakes with the co‐production group and sharing these experiences with the wider research team as points for reflection.

A comprehensive realist review was carried out using realist logic to provide an explanatory focus, resulting in the inclusion of 43 sources from the United Kingdom and the United States, with the potential for programme theory to be applicable to other international health and care research systems. Aligning CMOCs to the theoretical framework of existing MRTs created more certainty in causal explanations in relation to the research questions set out in the protocol and illustrates the relationships between them [1]. The realist approach is a strength, lending to creativity and searching for explanations that make sense of complex systems [38].

The findings were derived from the United Kingdom and the United States evidence following an inclusive search strategy, which may indicate less attention to the exclusion of people from BAFDC in health and care research outside the United Kingdom and the United States, warranting further investigation. Two searches were undertaken for pragmatic reasons, which may be viewed as a limitation. The exclusion criteria restricted the programme theory to evidence only relating to adults from BAFDC, not children. Most of the sources were qualitative [45, 46, 47, 48, 49, 51, 52, 53, 55, 56, 57, 61, 62, 64, 66, 68, 69, 70, 71, 78, 81, 82, 84], with some key documents from the grey literature [43, 44, 70, 74, 76, 80, 81]. The inclusion of grey literature is a strength, capturing the lived experiences as written by some Black individuals [43, 44, 74, 80]. Box 5 summarises the key evidence gaps from the realist review.

Box 5 **Evidence gaps.**

- How best to educate White researchers to critically reflect on their positionality and racial identity.
- How best to cultivate openness and spaces to facilitate conversations about race and White fragility with a view to changing behaviour.
- How best to create space to discuss health and care research with people from BAFDC.
- More needs to be understood about the mechanism of counter‐storytelling and how it could influence the inclusion and participation of people from BAFDC.
- Understanding more about how confident health and care researchers are at working with individuals from BAFDC, as well as their attitudes towards building relationships with people from BAFDC.
- Examples of authentic co‐production with people from BAFDC to demonstrate the value of PPI.

### 5.2 What This Study Adds

Although this realist review was undertaken from a UK perspective, its findings are likely to be applicable in many other settings because of the transferability of underlying mechanisms [23, 25]. The review addresses a significant gap in the evidence through postulating an understanding of how people from BAFDC are currently being included, or not, in health and care research. This knowledge is central to informing policy, service design and delivery, as well as understanding what is preventing progress and what may bring about progress. The programme theory asserts that there are key deficits in context at meso and macro levels of the health and care research system, which may be preventing change and progress from occurring.

### 5.3 Implications for Policy, Practice and Research

This review indicates that there are a number of important changes which are needed around aligning health and care research with the needs of people from BAFDC, investing in relationships, placing more value on PPI, understanding how to create a more open culture for dialogue about race and mandating the sharing of findings. It is acknowledged that in England, the NIHR are prioritising efforts around inclusion in health and care research, and it is hoped that this realist review will provide important evidence to support their focus [21].

The findings from this realist review are currently informing the co‐production of recommendations and guidance that will be published in an ensuing paper.

## 6 Conclusion

In conclusion, this review has addressed the need to synthesise evidence relating to the lack of inclusion and participation of people from BAFDC in health and care research. To develop a more inclusive research system *with* and *for* people from BAFDC, policymakers must endorse the importance of acknowledging structural, institutional and individual racism and encourage open discussion about the influence of such racism on inclusion and participation, as well as the importance of diverse teams. Hidden mechanisms such as productive ignorance, colour‐blindness and implicit and complicit bias may be considered uncomfortable to acknowledge. Greater awareness is needed to make progress in relation to inclusion and participation and minimise tension between people from BAFDC and the health and care research system. Practical measures by funders, ethics committees and health and care research organisations/institutions should consider investing in building trustworthiness and credibility with people from BAFDC, making relationships a priority to develop familiarity, which leads to more positive outcomes. Strong relationships with key gatekeepers may help to forge partnerships, but this requires funders to value long‐term investment in community engagement, supporting researchers to be present in BAFDC [59, 60, 66, 70, 73, 79, 80, 81, 83, 93]. This also requires organisations and the people within them to critically reflect on the ideologies, norms and cultures that exist in relation to anti‐racism and how they may share power and responsibility with people from BAFDC [44, 58, 60, 66, 73, 75].

Fundamental gaps in the evidence base indicate that the contextual and causal factors that influence inclusion and participation by people from BAFDC need to be explored further, as well as a deeper interrogation of the mechanisms that underlie implicit and complicit bias [1, 14]. A realist evaluation involving primary data collection from key individuals from around the health and care research system and people from BAFDC is underway and will further refine, refute and extend the programme theory from the realist review, develop co‐produced recommendations and a prototype for an intervention(s) to facilitate inclusion and participation of people from BAFDC.

## Author Contributions

**Eleanor Hoverd:** conceptualisation, investigation, funding acquisition, writing – original draft, methodology, validation, visualisation, software, formal analysis, project administration, data curation, writing – review and editing. **Sophie Staniszewska:** writing – review and editing, supervision, formal analysis, methodology, conceptualisation, funding acquisition, validation. **Jeremy Dale:** conceptualisation, funding acquisition, methodology, writing – review and editing, formal analysis, supervision, validation. **Dawn Edge:** conceptualisation, funding acquisition, methodology, writing – review and editing, formal analysis, supervision, validation. **Rachel Spencer:** conceptualisation, funding acquisition, writing – review and editing, formal analysis, supervision, validation, methodology. **Violet Effiom:** writing – review and editing, formal analysis, investigation, software, validation, conceptualisation, methodology. **Dionne Gravesande:** writing – review and editing, formal analysis, conceptualisation, methodology. **Lorna Hollowood:** conceptualisation, methodology, writing – review and editing, formal analysis. **Samantha Johnson:** validation, writing – review and editing, methodology. **Tony Kelly:** conceptualisation, methodology, writing – review and editing, formal analysis. **Roy McFarlane:** methodology, writing – review and editing, formal analysis. **Esther Mukuka:** conceptualisation, methodology, writing – review and editing, formal analysis. **Shane Ward:** conceptualisation, methodology, writing – review and editing, formal analysis.

## Ethics Statement

The authors have nothing to report.

## Consent

The authors have nothing to report.

## Conflicts of Interest

The authors declare no conflicts of interest.

## Supporting information

Supplementary File 1 GRIPP 2 checklist.

Supplemental File 2 Data extraction form.

Supplemental File 3Grey sources.

Supplemental file 4 search strategy.

Supplemental File 5 Document characteristics.

Supplmental file 6 CMOCs.

## Data Availability

Secondary data was used to support the findings of this study.
