# Supplementary material for: Understanding Inclusion and Participation of People From Black African Diaspora Communities in Health and Care Research: A Realist Review
Source: Health Expect. 2025 May 22;28(3):e70298. doi: 10.1111/hex.70298 (PMC12098309; doi:10.1111/hex.70298)
Supplement: Supplementary file 1 — GRIPP 2 checklist. [file HEX-28-e70298-s004.docx]

GRIPP2 short form

| Section and topic | Item | Reported on page No |
| --- | --- | --- |
| 1: Aim | Report the aim of PPI in the study | 85-88 |
| 2: Methods | Provide a clear description of the methods used for PPI in the study | 91-101, 110-116, 205-208 |
| 3: Study results | Outcomes—Report the results of PPI in the study, including both positive and negative outcomes | 164-165, 480-486,526-527 |
| 4: Discussion and conclusions | Outcomes—Comment on the extent to which PPI influenced the study overall. Describe positive and negative effects | 73-76, 93-102, 110-122,226-229, 519-527 |
| 5: Reflections/critical perspective | Comment critically on the study, reflecting on the things that went well and those that did not, so others can learn from this experience | 478-505 |

PPI=patient and public involvement
