# Supplementary material for: Understanding Inclusion and Participation of People From Black African Diaspora Communities in Health and Care Research: A Realist Review
Source: Health Expect. 2025 May 22;28(3):e70298. doi: 10.1111/hex.70298 (PMC12098309; doi:10.1111/hex.70298)
Supplement: Supplementary file 2 — Supplemental File 2 Data extraction form. [file HEX-28-e70298-s003.docx]

**Supplemental file 2: Data extraction form**

**Data Extraction Form and Appraisal form - REAL-BAFDC** (Rapid Realist Review of inclusion and participation of people from Black African Diaspora Communities in health and care research) **Model**

**(adapted from Rycroft- Malone et al 2012 and CARES Realist Methodology Training Jagosh 2022[144, 196] )**

**See Appendix A at the end of this document for guidelines on using the form.**

| **Full reference:**  Title:  Item type:  Country:  Publication date:  Authors:  Journal:  Access:  Quality:  Group being researched: |
| --- |
| **Research questions**   1. How do past and current historical, social and political contexts in health and care research impact upon inclusion and participation in health and care research for people from BAFDC? 2. What aspects of key contexts may foster inclusion in health and care research? 3. What are the mechanisms that influence participant decision making for BAFDC and how do they work? 4. How may an intervention that facilitates inclusion and participation for BAFDC “work”? |
| **Theory area 1 – Elements of candidacy that influence inclusion and participation in health and care research.** |
| 1. How do individuals from BAFDC identify themselves as candidates, or not candidates when invited to take part in health and care research[197]? An individual is a candidate if they see themselves according to the principles of candidacy – see point 2. [197]. |
|  |
| 1. What impact do the elements of candidacy (identification of candidacy, navigation of services, permeability of services, appearance at services, adjudication by healthcare professionals, offers of , resistance to the service, conditions at the community and macro level of a system) have on inclusion and the decision to participate in health and care research [197]? |
|  |
| 1. What are the:   – political  – economic  - environmental contexts  (operating conditions that influence accessibility) [198] like for BAFDC in health and care research? |
|  |
| 1. What impact does the interaction between the potential candidate and the person (e.g. researcher/health professional/community leader) have on inclusion and participation in health and care research[197]? |
|  |
| 5. Is the evidence provided in this theory area good and relevant enough to be included in the synthesis (consider the issues below, including any that are not mentioned here, but may be of importance)? |
|  |

| **Theory area 2 – Critical Race Theory and inclusion and participation of BAFDC in health and care research.** |
| --- |
| 1. What impact do social determinants of health, race, power and privilege have on inclusion and the participation in health and care research? |
|  |
| 1. What impact does racism have at an individual level and structural level in the health and care research system? |
|  |
| 1. Is the evidence provided in this theory area good and RELEVANT ENOUGH to be included in the synthesis (consider issues of recruitment approach/methods, settings, eligibility, sample size, data collection, data analysis and claims made)? |
|  |

| **Theory area 3 – Elements of Network theory that may influence inclusion and participation of BAFDC in health and care research.** |
| --- |
| 1. What impact do resource and information channels , or “network pipes” (relationships in a social space whether via social media, or face to face[199], have on the decision to participate in health and care research by BAFDC? And how does information flow through “network pipes”[200]? |
|  |
| 1. What impact does social influence have on the decision to participate in health and care research by BAFDC?[199, 201]? |
|  |
| 1. Is the evidence provided in this theory area good and relevant enough to be included in the synthesis (consider issues of recruitment approach/methods, setting, eligibility, sample size, data collection, data analysis and claims made)? |
|  |

| **Theory area 4 – Elements of Narrative theory that may influence inclusion and participation of BAFDC in health and care research.** |
| --- |
| 1. What impact do events whether past, or current, have on narrative around health and care research within BAFDC? |
|  |
| 1. How do individual, community and cultural narratives impact on inclusion and participation of BAFDC in health and care research? |
|  |
| 1. How do narratives shape the identity of BAFDC, their value systems and behaviours in relation to health and care research? |
|  |
| 4.Is the evidence provided in this theory area good and relevant enough to be included in the synthesis (consider issues of sample size, data collection, data analysis and claims made)? |
|  |
| **Theory area 5 – Social Dominance** |
| 1.What impact does institutional discrimination have on inclusion of BAFDC in health and care research[202]? |
|  |
| 1. What impact do legitimising myths e.g. stereotypes, attitudes, beliefs about BAFDC and social dominance orientation have on inclusion and participation of BAFDC in health and care research[202]? |
|  |
| 1. Is the evidence provided in this theory area good and relevant enough to be included in the synthesis (consider issues of recruitment approach, setting, eligibility, sample size, data collection, data analysis and claims made)? |
|  |
| 1. Is there evidence of particular theoretical perspective(s) that may impact on the effectiveness of a potential intervention? |
|  |
| 1. Is there evidence of contextual factors that may impact on the effectiveness of a potential intervention? |
|  |

| **Appraisal Assessment (section to be completed at the end of reviewing literature)** |
| --- |
| **Usefulness and relevance of this study is: (see definitions)**   \| High \| Moderate \| Low \| None \| \| --- \| --- \| --- \| --- \|   **Definition of the categories**  HIGH:  This category is for papers that have high relevance to the realist review. This means that the framing of the research and, the research questions are highly matched to the review questions, the empirical findings are clearly described and there is a rich description of the process and context that can greatly advance the theoretical output of the review. The paper is a ‘key informant’.  MODERATE:  This category is for studies that show a ‘moderately’ relevant framing of the primary research to the review theories. This may mean that the literature reports on an intervention that supports inclusion and participation in health and care research with Black individuals or describes middle-range theories that may inform the review even if there is no relevant empirical data from the paper to populate the CMO configurations, or has a few areas that are of interest even if it is not entirely clear whether they will be used in the synthesis.  LOW:  This category is for research that has met the selection criteria in terms of relevance to the review questions and the initial programme theories (or Mid-Range Theories) but is relatively thin on the description of context and mechanism. It is not placed in the exclusion category because it contains at least one idea or statement about the context, about the mechanisms or about conceptualising outcomes that can be used for refining the theory and building a CMO configuration. 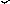 EXCLUDE:  This category is for a research paper that showed promise on reading the citation, but upon reading the full-text paper does not correspond to the review questions, does not have any content that corresponds to the initial programme theories (or MRT), or does not describe at all the context, or the mechanisms (or process). |

**Appendix A - Guidelines for using data extraction form – REAL BAFDC model.**

These guidelines are to help the reviewer(s) extract data that is relevant to the following research questions:

It may not be possible to record information in answer to all questions on the data extraction form, these are intended support identification of Context (C) Mechanism (M) Outcomes (O) Configurations (C) based upon theoretical areas. Evidence provided in the theory areas should be determined as good and relevant enough to be included in the synthesis (consider issues of sample size, data collection, data analysis and claims made, although do not exclude if poor quality but very relevant).Diverse data is important so the inclusion of grey literature e.g. conference abstracts, podcasts, social media posts etc…can be included if relevant to the development of programme theory[203].

Impact should be described as positive, negative, neutral, or unknown in regard to its bearing on inclusion, or participation.

Sources should be examined around the theory areas identified below. Details about the meaning behind the questions are provided, with cues to support data extraction, but this should not be restrictive. The REAL-BAFDC model is meant to provide a theoretical lens for this realist review.

**Theory area 1 – Elements of candidacy that influence inclusion and participation in health and care research.**

Examination of the literature/sources aims to identify and understand:

- Assessment of an individual’s eligibility for participation in health and care research

- Trust, information feedback, potential outcomes if one was to access a service

- The impact of historical contexts e.g., Windrush scandal,

- Knowledge (previous knowledge, how knowledge is obtained, needs improving etc.)

- What creates a sense of belonging

The key areas of candidacy include and are helpful to consider and examine:

- **Identification of candidacy**
  - Through what mechanism does an individual decide if they would like to participate in health and care research and how they legitimise themselves as a suitable candidate[197]
- **Navigation of services**
  - The knowledge an individual has of health and care research and how they reason what is needed to make contact and then access a health and care research study. This also means understanding the barriers to access e.g. financial implications, travel etc.. [197]
- **Permeability of services**
  - Through what mechanism does an individual take part in health and care research, noting any gate-keeping that prevents people from taking part; complications to taking part and cultural appropriateness of the service[197].
- **Appearance at services**
  - An individual’s ability to affirm whether they are a candidate and bring themselves to a health and care research study, or recruitment site and can articulate why they wish to take part in research[197].
- **Adjudication by healthcare professionals**
  - The judgement that health researchers, or healthcare professionals make about whether someone is a candidate for health and care research which may influence an individual, subsequently influencing the potential candidate’s movement through the research process, or through the health care service to get to take part in research. This may disadvantage some individuals due to discrimination, or because they are not served appropriately [197]
- **Offers of resistance to the service**
  - An individual might turn down an opportunity to take part in research throughout their healthcare journey, declining offers of appointments, invitations , or treatment offered in the study [197]
- **Conditions at the community and macro level of a system**
  - Are there available resources to help someone decide if they are a candidate for research and do relationships with health researchers, or health care professionals that develop through regular contact influence participation ?[197]

**Context**

Contextual factors can be identified at the individual, interpersonal, institutional and infrastructural levels of a system and therefore it would be useful to indicate which level the context is operating at. Contexts are the conditions that trigger mechanisms and may be key characteristics of circumstances that may support an intervention [203, 204].

- **Political contexts may include**: positions of power; institutional processes and power; ideological positions and how they affect the health and care research system impacting on individuals from BAFDC; distribution of money and resources[205]. May also include political priorities.
- **Economic contexts may include** economic factors that are related to health outcomes e.g., socio-economic status, current states of economies.
- **Environmental contexts may include** climate change; occupational health and how that impacts on decisions to take part in health and care research; culture and norms.

**Theory area 2 – Critical Race Theory and inclusion and participation of BAFDC in health and care research**

Examination of the literature/sources aims to identify and understand:

- **- Factors such as** housing conditions, education system, biases (implicit biases), racist beliefs, effects of racisms on inclusion and participation, discrimination, inclusion etc.

More specifically:

- Is there evidence of racism being normal and ingrained in research, how does it look normal to people, organisations, systems – this may be more notable in outcomes?
- Is White privilege taken for granted?
- How is racism masked?
- Is there evidence of storytelling by BAFDC that offer insightful contributions?
- Are there examples of race equality in operation when it benefits White people (interest convergence)?
- What are the structures , or relationships that maintain inequality[206]?

Describe impact as positive, negative, neutral or unknown.

**Theory area 3 – Elements of Network theory that may influence inclusion and participation of BAFDC in health and care research*.***

Examination of the literature/sources aims to identify and understand evidence in relation to**:**

- Resources and information channels ( mechanisms) can be informal , or formal and come from individuals, organisations, social networks, committees, community-based organisations [199].
- Social influence is a mechanism, as well as how individuals with status signal and approve things e.g. health and care research – these are key to identify [199].
- Relationships in networks can create responsibilities of loyalty e.g. conformity and have such influence that they can shield information, or fact[199].
- Social networks can lead to groupthink [199].
- How does this maintain the status quo – consider this at all levels of the system (micro, meso, macro)?

**Theory area 4 – Elements of Narrative theory that may influence inclusion and participation of BAFDC in health and care research.**

Examination of the literature/sources aims to identify and understand:

- Differences in perspectives
  - How these are communicated
  - How they support individuals’ identities, their values, beliefs and behaviours[207]
- acknowledgement of colonial history in regards to science and development of knowledge is critical and how , or if it is acknowledged[207].

**Theory area 5 – Social Dominance**

Examination of the literature/sources aims to identify and understand:

- Institutional discrimination e.g., how, or if institutions provide better access, or services to the dominant social group.
  - Indications of how it is disguised, or legitmised e.g. stereotypes - Black people are aggressive, White people are assertive , or countries asserting that they are democratic, not colonizing, rejecting institutional racism [202, 208], ?
- Consideration of assumptions (theoretical perspectives) about the current reality that may help to answer the research questions.
- Contextual factors that may impact effectiveness of a potential intervention. (This should become apparent through some of the previous questions but is helpful to double-check .)
