# Supplementary material for: Understanding Inclusion and Participation of People From Black African Diaspora Communities in Health and Care Research: A Realist Review
Source: Health Expect. 2025 May 22;28(3):e70298. doi: 10.1111/hex.70298 (PMC12098309; doi:10.1111/hex.70298)
Supplement: Supplementary file 3 — Supplemental File 3Grey sources. [file HEX-28-e70298-s006.docx]

Supplemental file 3: Grey sources searched

| National Institute for Health and Care Research (NIHR)  NHS Race and Health Observatory  Runnymede Trust  BRAP  The Health Foundation  The King’s Fund  Operation Black Vote  The Voice newspaper  African Voice newspaper  West Bromwich African Caribbean Resource Centre  Enfield Caribbean Association  CAHN (Caribbean and African Health Network)  Vanderbilt University Medical Centre, Office of Health Equity Anti-racism hub  CARE (Community, Access, Recruitment and Engagement) Center, Massachusetts General Hospital  Open Access Theses and Dissertations  Caribbean-studies@jiscmail.ac.uk  Caribbeanintelligence  Twitter, Facebook and YouTube |
| --- |
