# Supplementary material for: Understanding Inclusion and Participation of People From Black African Diaspora Communities in Health and Care Research: A Realist Review
Source: Health Expect. 2025 May 22;28(3):e70298. doi: 10.1111/hex.70298 (PMC12098309; doi:10.1111/hex.70298)
Supplement: Supplementary file 4 — Supplemental file 4 search strategy. [file HEX-28-e70298-s002.docx]

**Supplementary file 4: Search strategy process and searches**

Keywords comprised : research, inclusion, underrepresentation, clinical trials, participation and Black people [1].

Research and Academic Librarian (SJ) from Warwick Medical School (WMS) helped to develop the search strategy. There were no language limitations with documents included from the earliest date of databases. Search terms were piloted as detailed in the protocol[1].The co-production group felt it was important to capture international evidence due to the shared histories that people from BAFDC hold. Consequently, the search strategy reflected this. To ensure a level of preciseness , advice from SJ was to include a filter to MEDLINE and Embase searches , developed by the National Institute for Clinical Excellence (NICE) which was placed at the end of the search to capture UK evidence, reducing the number needed to read as well as identifying UK researchers working on research in other countries[42]. This was utilised only for the initial search as the iterative search was specifically to identify UK documents.

Database searches of the initial search and iterative search are shown below.

| **Initial search** |
| --- |
| **Medline** |
| 1        exp Clini  cal Trials as Topic/ or clinical trial*.mp.    1312219  2        exp Health Services Research/ or exp Biomedical Research/     435865  3        exp African Americans/      63032  4        black british.mp.      134  5        participation.mp. or Patient Participation/ or Community Participation/ or Stakeholder Participation/ or Social Participation/ 234944  6        inclusion.mp. 363848  7        recruitment.mp.       163464  8        (underrepresent* or under-represent*).mp.        21179  9        Black people.mp. or Black People/          41827  10       Afro-Caribbean.mp. 1316  11       BME.mp.      3459  12       BAME.mp.    385  13       1 or 2  1715031  14       6 or 7 or 8     541059  15       5 or 14          760230  16       exp United Kingdom/          393319  17       (national health service* or nhs*).ti,ab,in. 285610  18       (english not ((published or publication* or translat* or written or language* or speak* or literature or citation*) adj5 english)).ti,ab.          52462  19       (gb or "g.b." or britain* or (british* not "british columbia") or uk or "u.k." or united kingdom* or (england* not "new england") or northern ireland* or northern irish* or scotland* or scottish* or ((wales or "south wales") not "new south wales") or welsh*).ti,ab,jw,in.         2524313  20       (bath or "bath's" or (birmingham not alabama*) or ("birmingham's" not alabama*) or bradford or "bradford's" or brighton or "brighton's" or bristol or "bristol's" or carlisle or "carlisle's" or (cambridge not (massachusetts* or boston* or harvard*)) or ("cambridge's" not (massachusetts* or boston* or harvard*)) or (canterbury not zealand*) or ("canterbury's" not zealand*) or chelmsford or "chelmsford's" or chester or "chester's" or chichester or "chichester's" or coventry or "coventry's" or derby or "derby's" or (durham not (carolina* or nc)) or ("durham's" not (carolina* or nc)) or ely or "ely's" or exeter or "exeter's" or gloucester or "gloucester's" or hereford or "hereford's" or hull or "hull's" or lancaster or "lancaster's" or leeds* or leicester or "leicester's" or (lincoln not nebraska*) or ("lincoln's" not nebraska*) or (liverpool not (new south wales* or nsw)) or ("liverpool's" not (new south wales* or nsw)) or ((london not (ontario* or ont or toronto*)) or (("london's" not (ontario or ont or toronto*)) or manchester or "manchester's" or (newcastle not (new south wales* or nsw)) or ("newcastle's" not (new south wales* or nsw)) or norwich or "norwich's" or nottingham or "nottingham's" or oxford or "oxford's" or peterborough or "peterborough's" or plymouth or "plymouth's" or portsmouth or "portsmouth's" or preston or "preston's" or ripon or "ripon's" or salford or "salford's" or salisbury or "salisbury's" or sheffield or "sheffield's" or southampton or "southampton's" or st albans or stoke or "stoke's" or sunderland or "sunderland's" or truro or "truro's" or wakefield or "wakefield's" or wells or westminster or "westminster's" or winchester or "winchester's" or wolverhampton or "wolverhampton's" or (worcester not (massachusetts* or boston* or harvard*)) or ("worcester's" not (massachusetts* or boston* or harvard*)) or (york not ("new york" or ny or ontario* or ont or toronto*)) or ("york's" not ("new york" or ny or ontario* or ont or toronto*))))).ti,ab,in.          1814078  21       (bangor or "bangor's" or cardiff or "cardiff's" or newport or "newport's" or st asaph or "st asaph's" or st davids or swansea or "swansea's").ti,ab,in.  73333  22       (aberdeen or "aberdeen's" or dundee or "dundee's" or edinburgh or "edinburgh's" or glasgow or "glasgow's" or inverness or (perth not australia*) or ("perth's" not australia*) or stirling or "stirling's").ti,ab,in.     266882  23       (armagh or "armagh's" or belfast or "belfast's" or lisburn or "lisburn's" or londonderry or "londonderry's" or derry or "derry's" or newry or "newry's").ti,ab,in.         35355  24       (exp africa/ or exp americas/ or exp antarctic regions/ or exp arctic regions/ or exp asia/ or exp oceania/) not (exp great britain/ or europe/)     3393284  25       or/16-23        3168382  26       25 not 24      2997511  27       1 or 2  1715031  28       3 or 4 or 9 or 10 or 11 or 12          103826  29       5 and 28       2624  30       6 or 7 or 8     541059  31       5 or 30          760230  32       27 and 28 and 31    1316  33       26 and 32     35 |
| **EMBASE** |
| 1 clinical trials.mp. or "clinical trial (topic)"/ 571565  2 Health services research.mp. or health services research/ 43915  3 1 or 2 614850  4 Blacks/ or blacks.mp. 62822  5 African Americans.mp. or exp African American/ 131371  6 black british.mp. 294  7 4 or 5 or 6 186406  8 patient participation/ or participation.mp. or community participation/ or social participation/ 307358  9 3 and 7 and 8 827  10 inclusion.mp. 586250  11 recruitment.mp. 245216  12 (underrepresent* or under-represent*).mp. 31614  13 10 or 11 or 12 849875  14 8 or 13 1132273  15 3 and 7 and 14 1643  16 1 and 7 and 8 801  17 exp United Kingdom/ 515710  18 (national health service* or nhs*).ti,ab,in. 446363  19 (english not ((published or publication* or translat* or written or language* or speak* or literature or citation*) adj5 english)).ti,ab. 69108  20 or/17-19 933939  21 15 and 20 42  22 (gb or "g.b." or britain* or (british* not "british columbia") or uk or "u.k." or united kingdom* or (england* not "new england") or northern ireland* or northern irish* or scotland* or scottish* or ((wales or "south wales") not "new south wales") or welsh*).ti,ab,jw,in. 3994752  23 (bath or "bath's" or (birmingham not alabama*) or ("birmingham's" not alabama*) or bradford or "bradford's" or brighton or "brighton's" or bristol or "bristol's" or carlisle or "carlisle's" or (cambridge not (massachusetts* or boston* or harvard*)) or ("cambridge's" not (massachusetts* or boston* or harvard*)) or (canterbury not zealand*) or ("canterbury's" not zealand*) or chelmsford or "chelmsford's" or chester or "chester's" or chichester or "chichester's" or coventry or "coventry's" or derby or "derby's" or (durham not (carolina* or nc)) or ("durham's" not (carolina* or nc)) or ely or "ely's" or exeter or "exeter's" or gloucester or "gloucester's" or hereford or "hereford's" or hull or "hull's" or lancaster or "lancaster's" or leeds* or leicester or "leicester's" or (lincoln not nebraska*) or ("lincoln's" not nebraska*) or (liverpool not (new south wales* or nsw)) or ("liverpool's" not (new south wales* or nsw)) or ((london not (ontario* or ont or toronto*)) or (("london's" not (ontario or ont or toronto*)) or manchester or "manchester's" or (newcastle not (new south wales* or nsw)) or ("newcastle's" not (new south wales* or nsw)) or norwich or "norwich's" or nottingham or "nottingham's" or oxford or "oxford's" or peterborough or "peterborough's" or plymouth or "plymouth's" or portsmouth or "portsmouth's" or preston or "preston's" or ripon or "ripon's" or salford or "salford's" or salisbury or "salisbury's" or sheffield or "sheffield's" or southampton or "southampton's" or st albans or stoke or "stoke's" or sunderland or "sunderland's" or truro or "truro's" or wakefield or "wakefield's" or wells or westminster or "westminster's" or winchester or "winchester's" or wolverhampton or "wolverhampton's" or (worcester not (massachusetts* or boston* or harvard*)) or ("worcester's" not (massachusetts* or boston* or harvard*)) or (york not ("new york" or ny or ontario* or ont or toronto*)) or ("york's" not ("new york" or ny or ontario* or ont or toronto*))))).ti,ab,in. 3175674  24 (bangor or "bangor's" or cardiff or "cardiff's" or newport or "newport's" or st asaph or "st asaph's" or st davids or swansea or "swansea's").ti,ab,in. 129145  25 (aberdeen or "aberdeen's" or dundee or "dundee's" or edinburgh or "edinburgh's" or glasgow or "glasgow's" or inverness or (perth not australia*) or ("perth's" not australia*) or stirling or "stirling's").ti,ab,in. 439256  26 (armagh or "armagh's" or belfast or "belfast's" or lisburn or "lisburn's" or londonderry or "londonderry's" or derry or "derry's" or newry or "newry's").ti,ab,in. 61699  27 (exp africa/ or exp americas/ or exp antarctic regions/ or exp arctic regions/ or exp asia/ or exp oceania/) not (exp great britain/ or europe/) 3908704  28 or/17-27 8559048  29 28 not 27 4650344  30 1 or 2 614850  31 African Caribbean/ 3194  32 BME.mp. 2883  33 BAME.mp. 651  34 4 or 5 or 6 or 31 or 32 or 33 192289  35 8 and 34 4958  36 3 and 34 and 13 and 8 500  37 29 and 36 36 |
| **PsychINFO** |
| 1 clinical trials as Topic/ or clinical trial*.mp. 52102  2 Blacks/ or blacks.mp. 63518  3 Health Services Research.mp. 6507  4 1 or 3 58443  5 African Americans.mp. or exp African American/ 69556  6 black british.mp. 87  7 2 and 5 and 6 37  8 participation.mp. or Patient Participation/ or Community Participation/ or Stakeholder Participation/ or Social Participation/ 126217  9 inclusion.mp. 68543  10 participation.mp. 126217  11 2 and 4 and 10 141  12 (underrepresent* or under-represent*).mp. 9867  13 9 or 10 or 12 198415  14 recruitment.mp. 28267  15 13 or 14 223006  16 11 and 12 and 14 14 |
| **Web of Science** |
| Query link: https://www.webofscience.com/wos/woscc/summary/165d08fb-7eec-4e35-a3dd-6b111be8e250-f7a62633/relevance/1  199 results from Web of Science Core Collection for: black people and participation and clinical trials (All Fields) |
| **Race Relations Abstracts** |
| \| **Query** \| **Limiters/Expanders** \| **Last Run Via** \| **Results** \|  \| \| --- \| --- \| --- \| --- \| --- \| \| S9 \| S5 AND S6 AND S8 \| Expanders - Apply equivalent subjects \| Interface - EBSCOhost Research Databases \| 9 \| \| Search modes - Find all my search terms \| Search Screen - Advanced Search \| \|  \| Database - Race Relations Abstracts \| \| S8 \| S1 OR S2 OR S3 OR S4 \| Expanders - Apply equivalent subjects \| Interface - EBSCOhost Research Databases \| 26,701 \| \| Search modes - Find all my search terms \| Search Screen - Advanced Search \| \|  \| Database - Race Relations Abstracts \| \| S7 \| representation \| Expanders - Apply equivalent subjects \| Interface - EBSCOhost Research Databases \| 2,482 \| \| Search modes - Find all my search terms \| Search Screen - Advanced Search \| \|  \| Database - Race Relations Abstracts \| \| S6 \| participation \| Expanders - Apply equivalent subjects \| Interface - EBSCOhost Research Databases \| 3,240 \| \| Search modes - Find all my search terms \| Search Screen - Advanced Search \| \|  \| Database - Race Relations Abstracts \| \| S5 \| clinical trials or clinical research \| Expanders - Apply equivalent subjects \| Interface - EBSCOhost Research Databases \| 306 \| \| Search modes - Find all my search terms \| Search Screen - Advanced Search \| \|  \| Database - Race Relations Abstracts \| \| S4 \| black african \| Expanders - Apply equivalent subjects \| Interface - EBSCOhost Research Databases \| 8,065 \| \| Search modes - Find all my search terms \| Search Screen - Advanced Search \| \|  \| Database - Race Relations Abstracts \| \| S3 \| black caribbean \| Expanders - Apply equivalent subjects \| Interface - EBSCOhost Research Databases \| 794 \| \| Search modes - Find all my search terms \| Search Screen - Advanced Search \| \|  \| Database - Race Relations Abstracts \| \| S2 \| black british \| Expanders - Apply equivalent subjects \| Interface - EBSCOhost Research Databases \| 1,116 \| \| Search modes - Find all my search terms \| Search Screen - Advanced Search \| \|  \| Database - Race Relations Abstracts \| \| S1 \| black or african american or african-american or black american \| Expanders - Apply equivalent subjects \| Interface - EBSCOhost Research Databases \| 26,701 \| \| Search modes - Find all my search terms \| Search Screen - Advanced Search \| \|  \| Database - Race Relations Abstracts \| |
| **Sociological Abstracts** |
| (clinical trials) AND (blacks people) AND (Health Services Research) AND underrepresentation AND (adult participation) AND inclusion  66 |
| **University of West Indies Scholar** |
| 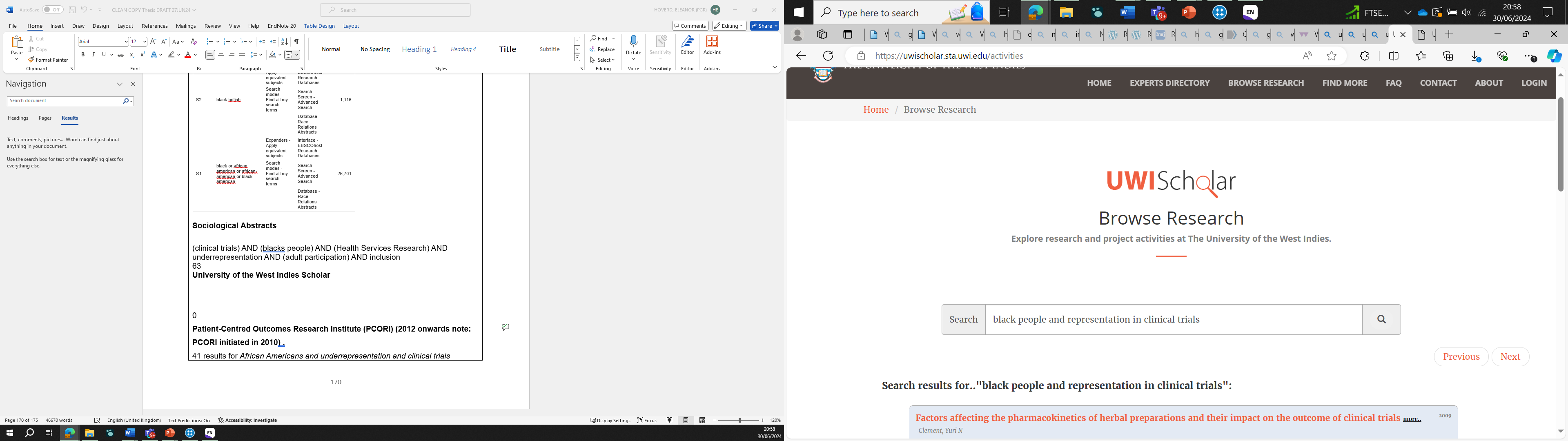0 relevant results |
| **Patient-Centred Outcomes Research Institute (PCORI) (2012 onwards - note: PCORI initiated in 2010).** |
| 41 results for *African Americans and underrepresentation and clinical trials* |

| **Iterative search** |
| --- |
| **Medline** |
| 1 exp "black or exp african people/ or exp black people/ 97488  2 exp "Ethnic and Racial Minorities"/ 563  3 ethnic minorit*.mp. 16053  4 1 or 2 or 3 111622  5 Research Design/ or Research Subjects/ or Biomedical Research/ or Genetic Research/ or Health Services Research/ or Interdisciplinary Research/ or Empirical Research/ or Social Validity, Research/ or Behavioral Research/ or Nursing Research/ or Community-Based Participatory Research/ or Rehabilitation Research/ or Fetal Research/ or Research/ 460578  6 exp Clinical Trial/ 978400  7 exp Clinical Trials as Topic/ 384334  8 clinical trial*.mp. 1177860  9 5 or 6 or 7 or 8 1984316  10 4 and 9 9351  11 (PPI or PPIE).mp. [mp=title, book title, abstract, original title, name of substance word, subject heading word, floating sub-heading word, keyword heading word, organism supplementary concept word, protocol supplementary concept word, rare disease supplementary concept word, unique identifier, synonyms, population supplementary concept word, anatomy supplementary concept word] 28573  12 exp Patient Participation/ 29527  13 public engagement.mp. 1724  14 exp Community Participation/ 48066  15 public participant involvement.mp. 0  16 "Patient and public involvement".mp. 1511  17 (public adj3 (invol* or engag* or participat*)).mp. [mp=title, book title, abstract, original title, name of substance word, subject heading word, floating sub-heading word, keyword heading word, organism supplementary concept word, protocol supplementary concept word, rare disease supplementary concept word, unique identifier, synonyms, population supplementary concept word, anatomy supplementary concept word] 10218  18 (Patient adj3 (involv* or engag* or participat*)).mp. [mp=title, book title, abstract, original title, name of substance word, subject heading word, floating sub-heading word, keyword heading word, organism supplementary concept word, protocol supplementary concept word, rare disease supplementary concept word, unique identifier, synonyms, population supplementary concept word, anatomy supplementary concept word] 54208  19 11 or 12 or 13 or 14 or 15 or 16 or 17 or 18 107589  20 10 and 19 395  21 Refusal to Participate/ 644  22 (underrepresent* or under-represent* or under represent*).mp. [mp=title, book title, abstract, original title, name of substance word, subject heading word, floating sub-heading word, keyword heading word, organism supplementary concept word, protocol supplementary concept word, rare disease supplementary concept word, unique identifier, synonyms, population supplementary concept word, anatomy supplementary concept word] 19779  23 Patient Selection/ or recruitment.mp. 223659  24 retention.mp. 230655  25 21 or 22 or 23 or 24 462786  26 10 and 25 1543  27 20 or 26 1770  28 UK.mp. or United Kingdom/ 335061  29 27 and 28 17 |
| **EMBASE** |
| 1 "black or african american"/ or exp african people/ or exp black people/ 185166  2 exp "ethnic and racial minorities"/ 191759  3 ethnic minorit*.mp. 20575  4 1 or 2 or 3 371745  5 Research Design/ or Research Subjects/ or Biomedical Research/ or Genetic Research/ or Health Services Research/ or Interdisciplinary Research/ or Empirical Research/ or Social Validity, Research/ or Behavioural Research/ or Nursing Research/ or Community-Based Participatory Research/ or Rehabilitation Research/ or Fetal Research/ or Research/ 2793740  6 exp Clinical Trial/ 1855602  7 exp Clinical Trials as Topic/ 442999  8 clinical trials*.mp. 472732  9 5 or 6 or 7 or 8 5181996  10 4 and 9 43764  11 (PPI or PPIE).mp. [mp=title, abstract, heading word, drug trade name, original title, device manufacturer, drug manufacturer, device trade name, keyword heading word, floating subheading word, candidate term word] 43497  12 exp Patient Participation/ 35767  13 public engagement.mp. 1845  14 exp Community Participation/ 4917  15 "public participant invovlement".mp. 0  16 (public adj3 (invol* or engag* or participat*)).mp. [mp=title, abstract, heading word, drug trade name, original title, device manufacturer, drug manufacturer, device trade name, keyword heading word, floating subheading word, candidate term word] 12590  17 (Patient adj3 (involv* or engag* or participat*)).mp. [mp=title, abstract, heading word, drug trade name, original title, device manufacturer, drug manufacturer, device trade name, keyword heading word, floating subheading word, candidate term word] 74798  18 11 or 12 or 13 or 14 or 15 or 16 or 17 131624  19 10 and 18 705  20 UK.mp. or United Kingdom/ 618754  21 19 and 20 16 |
| **PsychINFO** |
| 1 (Black or African or Black British or Black Caribbean or Black African).mp. [mp=title, abstract, heading word, table of contents, key concepts, original title, tests & measures, mesh word] 112138  2 (Ethnic and racial minorities).mp. [mp=title, abstract, heading word, table of contents, key concepts, original title, tests & measures, mesh word] 886  3 ethnic minorit*.mp. 14516  4 1 or 2 or 3 124104  5 Research Design/ or Research Subjects/ or Biomedical Research/ or Genetic Research/ or Health Services Research/ or Interdisciplinary Research/ or Empirical Research/ or Social Validity, Research/ or Behavioral Research/ or Nursing Research/ or Community-Based Participatory Research/ or Rehabilitation Research/ or Fetal Research/ or Research/ 82882  6 exp Clinical Trial/ 13689  7 clinical trial*.mp. 53709  8 5 or 6 or 7 134531  9 4 and 8 2486  10 (PPI or PPIE).mp. [mp=title, abstract, heading word, table of contents, key concepts, original title, tests & measures, mesh word] 3157  11 exp Patient Participation/ 3199  12 public engagement.mp. 616  13 (public adj3 (invol* or engag* or participat*)).mp. [mp=title, abstract, heading word, table of contents, key concepts, original title, tests & measures, mesh word] 5531  14 11 or 12 or 13 8587  15 9 and 14 35  16 UK.mp. 44654  17 United Kingdom.mp. 16048  18 16 or 17 55536  19 15 and 18 56 |
| **Web of Science** |
| Black people or ethnic minorities and Patient Public Involvement and clinical trials + United Kingdom  61 citations |
| **Race Relations Abstracts** |
| \| **#** \| **Query** \| **Limiters/Expanders** \| **Last Run Via** \| **Results** \| \| --- \| --- \| --- \| --- \| --- \| \| S7 \| S1 AND S4 \| Expanders - Apply equivalent subjects \| Interface - EBSCOhost Research Databases \| 4 \| \| Search modes - Find all my search terms \| Search Screen - Advanced Search \| \|  \| Database - Race Relations Abstracts \| \| S6 \| S1 AND S4 \| Expanders - Apply equivalent subjects \| Interface - EBSCOhost Research Databases \| 2 \| \| Search modes - Find all my search terms \| Search Screen - Advanced Search \| \|  \| Database - Race Relations Abstracts \| \| S5 \| research design \| Expanders - Apply equivalent subjects \| Interface - EBSCOhost Research Databases \| 1,072 \| \| Search modes - Find all my search terms \| Search Screen - Advanced Search \| \|  \| Database - Race Relations Abstracts \| \| S4 \| patient public involvement \| Expanders - Apply equivalent subjects \| Interface - EBSCOhost Research Databases \| 6 \| \| Search modes - Find all my search terms \| Search Screen - Advanced Search \| \|  \| Database - Race Relations Abstracts \| \| S3 \| bme or black minority ethnic or ethnic group or bame \| Expanders - Apply equivalent subjects \| Interface - EBSCOhost Research Databases \| 7,818 \| \| Search modes - Find all my search terms \| Search Screen - Advanced Search \| \|  \| Database - Race Relations Abstracts \| \| S2 \| black british \| Expanders - Apply equivalent subjects \| Interface - EBSCOhost Research Databases \| 1,116 \| \| Search modes - Find all my search terms \| Search Screen - Advanced Search \| \|  \| Database - Race Relations Abstracts \| \| S1 \| black people \| Expanders - Apply equivalent subjects \| Interface - EBSCOhost Research Databases \| 5,238 \| \| Search modes - Find all my search terms \| Search Screen - Advanced Search \| \|  \| Database - Race Relations Abstracts \| |
| **Sociological Abstracts** |
| ethnic minorities and research design  Applied filters United Kingdom – UK  48 records |
