# Supplementary material for: Understanding Inclusion and Participation of People From Black African Diaspora Communities in Health and Care Research: A Realist Review
Source: Health Expect. 2025 May 22;28(3):e70298. doi: 10.1111/hex.70298 (PMC12098309; doi:10.1111/hex.70298)
Supplement: Supplementary file 5 — Supplemental File 5 Document characteristics. [file HEX-28-e70298-s005.docx]

| **Reference** | **Year** | **Country** | **Type of Paper** | **Aim** | **Methods** | **Participants** |
| --- | --- | --- | --- | --- | --- | --- |
| Andrews N et al Intersectionality in the Liminal Space: Researching Caribbean Women's Health in the UK Context | 2019 | UK | Research | To develop applied health research methodology with African Caribbean women. | Through natural talk in liminal spaces, data was collected on the conversations Black Caribbean women had about health. | African Caribbean women in liminal spaces (e.g. hairdressing salons) Birmingham, England, UK. |
| Aguiar  The Black patient experience is still negative | 2023 | UK | Newspaper article | To share the impact of healthcare experiences of Black people in the NHS. | Media report | Black adults using the healthcare services in the NHS |
| Andrews K Aggrey Burke was the NHS’s first Black consultant psychiatrist. Rather than becoming a pillar of the establishment, he was forced to challenge it when he saw how other people of colour were treated | 2022 | UK | Newspaper article | To share the experiences of Black people and how they are treated. | Media report | Black adults and their lived experiences in the UK. |
| Blakey H Participation why bother? The views of Black and Minority Ethnic mental health service users on participation in the NHS in Bradford. Report of a community research process undertaken by the International Centre for Participation Studies, University of Bradford and Sharing Voices (Bradford). | 2005 | UK | Working paper | To understand participation of Black and minority ethnic people on participation in the NHS | Community research process  5 community workshops | African Caribbean and South Asian adults using mental health services in the NHS. Bradford, England. |
| Brewer et al  Preventing cardiovascular disease: Participant perspectives of the FAITH! Program | 2019 | US | Research | To understand the perceptions of African American individuals around culturally tailored education programmes delivered through a community participatory approach | Discussion groups, survey, (intervention 8, 90-minute education sessions held bi-weekly at a church) | 37 African Americans (age 18 yrs and older) who were part of church congregations in Minnesota, US. |
| Bridges et al  Talking trials: An arts-based exploration of attitudes to clinical trials amongst minority ethnic members of the South Riverside Community of Cardiff | 2023 | UK | Research | To explore the perceptions of individuals from ethnic minority backgrounds about research. | Art-based project using a co-production approach to engage with individuals from ethnic minority backgrounds. | 13 individuals in Cardiff from diverse ethnic backgrounds: Indian (3), Pakistani (1), Somali (2), Bissau-Guinean (4), Sudanese (2), Italian (1) |
| Bruce et al  Knowledge and perceptions about clinical trial participation among African American and Caucasian College students | 2014 | US | Research | To investigate the knowledge and perceptions of undergraduate and postgraduate students about participation in clinical trials and how that may vary according to race/ethnicity. | Cross-sectional survey | Students between 18-35 years of age from a public college (predominantly serving ethnic minorities) and a private college (predominantly White students).  171 African American students and 119 White students. |
| Cocroft S et al  Racially diverse participant registries to facilitate the recruitment of African Americans into presymptomatic Alzheimer’s disease studies | 2020 | US | Research | To gain insight into recruitment strategies to increase representation of African Americans in Alzheimer’s research | Systematic review | African American populations that are part of the Alzheimer’s Disease Prevention Registry (ADPR), North Carolina, US. |
| Cunningham-Erves J et al  A Pilot Study of a Culturally Appropriate, Educational Intervention to Increase Participation in Cancer Clinical Trials among African Americans and Latinos | 2021 | US | Research | To investigate the effect of a culturally appropriate educational program on knowledge and trust in medical researchers and their intent to take part in research. | Pilot study: Video, town halls, pre-post survey to evaluate intervention using convenience sampling. | 116 African Americans and 82 Latino adult populations who went to “townhalls” in Tennessee, US. |
| Cunningham-Erves et al  Formative research to design a culturally appropriate cancer clinical trial education program to increase participation of African American and Latino communities | 2020 | US | Research | To develop a community engaged educational research programme that improves, knowledge, awareness and understanding of clinical trials to do with cancer. | Qualitative. Initial “town hall” conversational sessions were held and then focus groups to iteratively inform an educational programme. Purposive sampling methods used. | 10 focus groups of African American and Latino populations aged 18 or older. 78.8% female, 21.2% male, 36.5% Black or African American. Mean age 42 years. |
| Dobransky-Farsiska D et al Developing a Community-Academic Partnership to Improve Recognition and Treatment of Depression in Underserved African American and White Elders | 2009 | US | Research | To develop a community-academic partnership with African American elders. | CBPR approach building a communications platform, fostering relationships, assessing needs, meeting needs, maintenance of partnership. | 26 Community partners who serve African American and White older people, Pennsylvania. |
| Ekezie et al  Patient and public involvement for ethnic minority research: an urgent need for improvement | 2021 | UK | Commentary | Provide recommendations about how to involve Black, Asian and other ethnic minority populations in health and care research. | Publication to disseminate recommendations | Black, Asian and other ethnic minority populations as public contributors, UK. |
| Eliacin J et al  Factors influencing the participation of Black and White Americans in Alzheimer’s disease biomarker research | 2022 | US | Research | To better understand the perspectives in relation to participating in Alzheimer’s Disease research | Individual interviews and focus groups | 32 participants formed three groups of Black, or African American and White participants with a mixture of experience of taking part in research related to Alzheimer’s Disease, Indiana, US. Aged 55yrs or older. |
| Farooqi et al Developing a toolkit for increasing the participation of black, Asian and minority ethnic communities in health and social care research | 2022 | UK | Research | To develop an evidence-based toolkit for supporting researchers to recruit individuals from Black, Asian and minority ethnic groups. | Literature review, focus groups workshops and communication with participants. | Four focus groups of researchers (n=21) and individuals from Black, Asian and minority ethnic backgrounds (n=14), over 18 yrs old. Leicester, England, UK. |
| Ferre et al  The Healthy African American Families (HAAF) Project: From Community-Based Participatory Research To Community partnered Participatory Research | 2010 | US | Research | To share knowledge about how a community partnered reach approach works. | Descriptive methods of building and partnering with African American communities to shift power to communities in the development of research. | African American communities that are part of the Healthy African American Families project, Los Angeles, US. |
| Ford M et al Unequal Burden of Disease, Unequal  Participation in Clinical Trials: Solutions from African American and Latino Community Members | 2013 | US | Research | To identify solutions to participation in research by African Americans and Latino communities. | Focus groups identified through a marketing company, word of mouth, community adverts, conducted by ethnically matched researchers. Content analysis was undertaken. | Six homogenous focus groups or 57 adults, 32 African Americans, 25 Latinos aged 50 yrs and older. South Carolina, US. |
| Fouad M et al  Patient Navigation Model to Increase Participation of African Americans in Cancer Clinical Trials | 2016 | US | Research | To understand the impact of patient navigators on participation in cancer clinical trials with African Americans. | Patient navigators trained to support patients to navigate access to cancer clinical trials. | 424 African American patients with cancer. 304 who were eligible, took part in a trial and 272 received patient navigation Alabama, US. |
| Gilmore-Bykovskyi A et al Traversing the Aging Research and Health Equity Divide: Toward Intersectional Frameworks of Research Justice and Participation | 2021 | US | Discussion paper | To examine the intersectional reasons that cause health inequities in aging research amongst people from African American and other ethnic minority groups. | Discussion of frameworks and theoretical approaches to supporting inclusion of people from minoritised groups in ageing research. | African American adults and other minority ethnic groups, US. |
| Goff L et al Healthy Eating and Active Lifestyles for Diabetes (HEAL-D): study protocol for the design and feasibility trial, with process evaluation, of a culturally tailored diabetes self-management programme for African-Caribbean communities | 2019 | UK | Research (protocol) | Protocol To develop and evaluate the implementation of an I intervention to support type 2 diabetes self-management with people from Black African and Black Caribbean backgrounds and test its feasibility. | To describe the protocol for development of the intervention through focus groups, interviews and co-design workshops, followed by a feasibility study to test the intervention. | Black African and Black Caribbean adults in London with type 2 diabetes, UK. |
| Griffith et al Determinants of Trustworthiness to Conduct Medical Research: Findings from Focus Groups Conducted with Racially and Ethnically Diverse Adults | 2019 | US | Research | To describe how racially and ethnically diverse research participants feel makes researchers and institutions trustworthy. | Criterion sampling strategy of African American, Latinx and White adults in 7 focus groups. Conducted at community centers. | 57 adults from African American (42.1%), Latinx (36.8%) and White (19.3%) communities, Tennessee, US. |
| Halvorsud et al  Identifying evidence of effectiveness in the co-creation of research: a systematic review and meta-analysis of the international healthcare literature | 2019 | UK | Research | To investigate the evidence, gap around the effectiveness of co-production in international health research | Systematic review of the literature of 26 primary studies. | Ethnic minority adult populations in the UK who participated in primary research. |
| Hernandez N et al African American Cancer Survivors’ Perspectives on Cancer Clinical Trial Participation in a Safety-Net Hospital: Considering the Role of the Social Determinants of Health | 2021 | US | Research | To assess the facilitators and barriers to participation by African Americans in cancer clinical trials. | Focus groups (3) with 25 participants. Thematic analysis conducted with key themes around: lack of understanding; perceptions and fears of cancer trials, preferred role of patient navigator. | 25 African Americans with past or current cancer (18-75 yrs old), Atlanta, Georgia, US. |
| Iyizoba-Ebozue et al 2022 Reflection on Black and ethnic minority participation in clinical trials | 2022 | UK | Editorial | Reflection on the participation of Black and ethnic minority populations in health and care research | Reflective piece | Black and ethnic minority populations in the UK. |
| Jones et al Participation in Action: The Healthy African American Families Community Conference Model | 2010 | US | Research | To describe an overview of the Healthy African American Families (HAAF) community engagement conference model. | Narrative of HAAF and how participatory research is encouraged, as well as the dissemination findings and action following this. | African American adults from different socioeconomic classes and processions and have taken part in research or service delivery programmes and are part of the Healthy African Families project, Los Angeles, US. |
| Kennedy et al Challenging Assumptions About African American Participation in Alzheimer’s Trials | 2017 | US | Research | To undertake a comprehensive investigation of African American participation in Alzheimer’s disease trials and to understand the effects on increasing enrolment on participant outcomes. | Meta-analysis of Alzheimer’s Disease Cooperative Study ad Alzheimer’s Disease Neuroimaging Initiative. | 5,164 African American and White adults from the Alzheimer’s Disease Cooperative Study and Alzheimer’s Disease Neuroimaging Initiative, US. |
| Langford et al Development of a Plain Language Decision Support Tool for Cancer Clinical Trials: Blending health literacy, academic research, and minority patient perspectives | 2020 | US | Research | To develop a decision support tool for cancer patients from ethnic minority backgrounds. | Development of a web-based decision support tool through undertaking a literature review; qualitative interviews (n=45); review of a telephone survey (n=1100); user experience and acceptability data capture (n=9) | Black and Latino cancer patients from Southeast Florida. 15 Black adults, 30 Latino adults interviewed; 3 Black and 6 Latino adults took part in usability testing. |
| Lincoln et al  Fundamental causes of barriers to participation in Alzheimer’s clinical research among African Americans | 2021 | US | Research | To undertake qualitative exploration of the barriers and causes of participation in health research by African Americans in Alzheimer Disease trials | Qualitative research. 5 focus groups conducted. Thematic analysis | 44 African American men and women (aged 50yrs older) from two senior centres and two African American social clubs in the western US. (13 males, 31 females). |
| Liu J et al Achieving Ethnic Diversity in Trial Recruitment | 2011 | UK | Opinion piece | To discuss the lack of clarity around effective recruitment with people from ethnic minority populations in the UK. | To share and discuss strategies that may work as well as those that don’t in relation to recruitment with people from ethnic minority groups. | African American, Hispanic/Latino and Asian adult populations, UK. |
| Marcu A et al Adapting a breast cancer early presentation intervention for Black women: A focus group study with women of Black African and Black Caribbean descent in the United Kin | 2021 | UK | Research | To understand how best to adapt a breast cancer booklet to promote awareness, through PPI. | Focus groups (2-8 participants) with Black African and Black Caribbean women. Recruited via purposive and snowball sampling. Framework analysis. | 22 Black African and Black Caribbean women, five treated for breast cancer, Surrey. |
| Masood et al Synthesis of researcher reported strategies to recruit adults of ethnic minorities to clinical trials in the United Kingdom: A systematic revie | 2019 | UK | research | To identity recruitment strategies targeting Black, Asian and other ethnic minority communities. | Systematic review | Black, Asian and other ethnic minority populations. |
| Moreno-John et al Ethnic Minority Older Adults  Participating in Clinical Research | 2004 | US | Research | To discuss the under-representation of ethnic minority populations in health and care research | A review of the literature (21 studies included) around mistrust and health and care research | African American, Latino and American Indian adults in the UK. |
| Motune V  ‘Racist’ professors slammed after being accused of treating Africans like guinea pigs. | 2020 | UK | Newspaper article | To describe the effect of racist language and views regarding a media story during the COVID-19 pandemic and the impact this has on Black communities | Media report | Author is a newspaper journalist referring to people from Africa. |
| Mumba et al  African Americans in Research in the Era of Precision Medicine: An All of Us Research Program Initiative. | 2020 | US | Research | To increase awareness of a program called All of Us Research program aimed at engaging African Americans with discussion about research | Quasi-experimental study. Single group design. Pre/post surveys. Participants were recruited via purposive sampling and snowball sampling. | 70 adult African Americans from African American communities in rural and urban in Alabama, US. |
| NIHR Race Equality Framework | 2022 | UK | Framework | To develop a self-assessment tool for supporting organisations to improve race equity through PPI | 3 consultation events were held with Black African, Black Caribbean and Asian communities | Black African, Black Caribbean and Asian communities in the UK. |
| Parker et al  Use of critical race theory to inform the recruitment of Black/African American Alzheimer’s Disease caregivers int community-based research | 2022 | US | Research | Exploration of CRT and how it can be applied to strategies for improve participation of Black people in research. | Case exemplars of how storytelling, race consciousness and praxis can be used to develop culturally informed strategies for recruitment | Black and African Americans who are caregivers for people with Alzheimer’s disease, Maryland, US. |
| Robins-Sadler et al  Recruiting Research Participants at Community Education Sites | 2005 | US | Research | To understand if community-based education sites and community educators serving African American communities may improve recruitment to research. | Cluster recruitment through the marriage of community educators, researchers, and community sites. | 378 participants (from African American communities). (130 male, age 18-73yrs old); (248 female, age 18-81 yrs old). California, US. |
| Stockdill et al African American Recruitment in Early Heart Failure Palliative Care Trials: Outcomes and Comparison with the ENABLE CHF-PC Randomized Trial | 2023 | US | Research | To examine the recruitment of African Americans into palliative care trials. | Descriptive analysis of a RCT to compare racial and patient characteristics through the recruitment process of palliative care trials. | 415 participants (226 = African American) aged 50 yrs or older, diagnosed with Heart Failure (NYHA III/IV); 53.7% male, mean age 63.8 yrs old, Alabama, US. |
| Udonya I  Black people are not your guinea pigs | 2020 | UK | Newspaper article | To share opinion around the impact of historical abuse and continued harmful narratives towards Black people in relation to scientific research | Media report to voice concerns of Black people around the history of harmful, abusive and exploitative treatment | Author writes of Black people as users of healthcare systems and the stereotypes and harmful narratives about Black people. |
| Ward and Gardiner  Black, Covid and in Lockdown: In Our Own Words | 2021 | UK | Community-led research | To understand the experiences of people from BAFDC in the West Midlands following COVID-19 and lockdown. | Survey and telephone interviews, focus groups. | 119 adults from Black African Diaspora Communities in the West Midlands. |
| Wenzel et al  A Model of Cancer Clinical Trial Decision-making Informed by African American Cancer Patients | 2015 | US | Research | To understand the research decision-making of African American cancer patients | Qualitative research. 7 focus groups. | 32 African American cancer patients over age 18, diagnosed with breast, prostate or colorectal cancer, Maryland, US. |
| Wilson P and Mavhandu-Mudzusi H Working in partnership with communities to improve health and research outcomes. Comparisons and commonalities between the UK and South Africa | 2019 | UK | Development paper | To share best practice of PPI of a South African community engagement project and examples of PPI in English primary and community care. | Analysis of frameworks for partnership working and mapping of common enablers and barriers to partnership working within different contexts with a focus on decolonisation. | Broad range of populations (including teachers, university students, academics from South Africa and UK. (social justice orientated approaches) |
| Williams et al  Barriers and Facilitators of African American Participation in Alzheimer’s Disease Biomarker Research | 2010 | US | Research | To investigate the barriers and facilitators of Alzheimer’s disease research participation by African Americans | Qualitative research. 11 focus groups. Convenience sampling via from Washington University Alzheimer’s Disease Research Centre, African American newspaper, flyers at health centres and community contacts. | 70 African Americans (73% female), mean age 52 years who had previously taken part in research., Washington, US. |
| Yancey et al  Effective recruitment and retention of minority research participants | 2006 | US | Research | To discuss the evidence in relation to the underrepresentation of African Americans and other ethnic minority groups in research. | Literature review of 75 studies, mostly descriptive. | African American, Latino, Native American Indian populations from the US. |
