# Supplementary material for: Understanding Inclusion and Participation of People From Black African Diaspora Communities in Health and Care Research: A Realist Review
Source: Health Expect. 2025 May 22;28(3):e70298. doi: 10.1111/hex.70298 (PMC12098309; doi:10.1111/hex.70298)
Supplement: Supplementary file 6 — Supplmental file 6 CMOCs. [file HEX-28-e70298-s001.docx]

| **Health and care research as a White space** | |
| --- | --- |
| ***Recruitment strategies led through a White-dominant culture (Initial Programme Theory (IPT) 1)*** | |
| **CMOC1 Recruitment targets perpetuate inequalities**  Recruitment targets for health and care research studies prioritise speed and high enrolment **(context)** which drive recruitment strategies to achieve high recruitment targets. This may be due to health and care research often being led by a White-dominant culture **(contextual influence)** whereby researchers and funding often go to White researchers and so individuals from BAFDC feel that health and care research is benefiting White populations **(mechanism)** and is focussed on financial gain and not about caring for their health. The influences of a mainly White culture means that recruitment strategies may end up not meeting the needs of individuals from under-served communities **(outcome 1)** like BAFDC due to the pressure **(mechanism)** of achieving higher recruitment targets and taking research to locations where it may be viewed as easier to recruit from, fuelling a “hard to reach” narrative **(mechanism)**. | [49,52,55-66,70] |
| ***Social and institutional conditions that affect who takes part (IPT2)*** | |
| **CMOC2 Racialised hegemony stifles inclusion**  Existing conditions within the health and care research system include a scientific workforce that lacks ethno-racial diversity **(context)** and visibly normalises participation as something that White people do. Behavioural asymmetry **(mechanism)** influences the candidacy of people from BAFDC. There is a lack of response to the needs of people from BAFDC due to the privileges upheld by racialised hegemony whereby racial categories are used to maintain power and dominance **(mechanism)**.This leads to the development of policies that are based upon the needs of White populations and may exclude people from BAFDC **(outcome 1)**.This results in inter-racial tensions **(outcome 2)** due to a lack of improvement in the wider social determinants of health. | [59,61,70,76,77,85] |
| **CMOC3 Homogeneity of funding**  There is a lack of racial balance for those who are awarded grants, by funders of health and care research **(context).** Funding tends to be unequally awarded **(mechanism)** to White researchers due to structural barriers and processes that may affect the chances of individuals from BAFDC having the same opportunities and access to the same networks. Researchers from ethnic minority groups are also less likely to receive funding for research grants and often receive less funding , when awarded grants, limiting diversity and representation **(outcome 1)**. This may make individuals from BAFDC feel like health and care research is not for them **(mechanism)** because those conducting research do not look like them and there is less understanding of their needs due to a lack of cultural awareness by a predominantly White scientific workforce **.**This results in less trust in the scientific workforce **(outcome 2)** by individuals from BAFDC, as well as a health and care research system that lacks diversity in its workforce and in the populations which take part. | [52,61,63] |
| ***Blame rhetoric (IPT3)*** | |
| **CMOC4 Cultural norms can influence narratives about under-representation**  Oppressive practices and norms **(context)** such as harmful narratives and scientific knowledge that uphold deficit thinking, or ideas about individuals from BAFDC that silences and misrepresents their lived experience and histories **(outcome 1)** can be exposed through the use of storytelling, or counter-storytelling **(mechanism)**. Counter-storytelling by people from BAFDC can counteract deficit narratives and harmful ideas, stereotypes and assumptions about BAFDC, building trust and helping to achieve social justice **(outcome 2)**. | [46,61,66,69,71,78,86] |
| **CMOC5 Placing the blame and responsibility on individuals from BAFDC to improve representation**  The conditions within the health and care research system **(context)** for example, the distribution of power and privilege is mainly amongst a White scientific/research workforce and thus scientific publications, narratives and cultures that exist have been written and established by a predominantly White workforce. Whiteness **(contextual influence)** upholds a blame rhetoric **(mechanism)** that is a result of racial bias and stereotypes e.g. lack of willingness to participate and people from BAFDC feel burdened with developing trust **(mechanism).**This justifies the marginalisation of people from BAFDC **(outcome)** through removing responsibility from the health and care researchers and placing the burden on Black communities to “trust” health and care research and researchers. | [49,61,70,73-75,81-83] |
| **Trust deficit: the expansiveness of broken trust** | |
| ***Insensitivity to Black suffering, disregard for Black lives (IPT4)*** | |
| **CMOC6 Influence of racism and historical ill-treatment of individuals from BAFDC on the decision to take part**  There is a continuum of hurt and unjust treatment towards people from BAFDC **(context)** that is a result of historical medical abuse and experimentation, exploitation, racism, and ongoing cultural trauma, that continues in the present. Racial discrimination and racism are so deeply entrenched within the UK culture and society does not always “see” these mechanisms if they are hidden in policies and practices, or, society does “see” them so frequently, that they do not consider how they may be affecting Black people’s lives because it has become a regular occurrence and is not spoken about openly **(mechanism)**. As a consequence, people from BAFDC do not feel they are given opportunities to be listened to **(mechanism),** involved in, or valued in healthcare **(context)** , or health and care research and White health and care researchers do not openly talk about the impact of racism because they feel afraid to **(mechanism)** as it may feel uncomfortable, they may fear repercussions, they may fear being accused of racism . This colourblind approach creates tension and perpetuates racism, ignoring the existence and reality of racism in wider society, health care and health and care research **(outcome 1)** .Colourblindness can lead to an ignorance of race-based differences and means that White people do not know how to talk about race **(outcome 2)**. | [46,54,56,58-59,63,71,74,85] |
| **CMOC7 Trust and confidence in health and care researchers and research institutions**  Health and care researchers and research institutions who are lacking in credibility **(context)** with Black communities may not be trusted. “Word of mouth” **(mechanism)** within Black communities and interactions with and the familiarity of health and care researchers **(mechanism)** to people from BAFDC leads to either trust and confidence in them and research **(outcome 1)** , or cause for concern **(outcome 2).** | [56-59,60,61,63,65,76,84,86] |
| **CMOC8 Competencies matter**  Inter-relationships between health and care researchers and individuals from BAFDC **(context)** requires cultural humility and a desire to tailor approaches to meet the needs of individuals from BAFDC .  A working partnership **(mechanism)** between health care researchers and individuals from BAFDC whereby researchers have developed cultural humility and value the lived experience and expertise of individuals from BAFDC as well as invest in additional resources required to work together as well as identifying benefits to the community **(mechanism)** makes individuals from BAFDC feel valued and cared about **(mechanism)**.This results in trust and respect between health and care researchers and people from BAFDC **(outcome)**. | [46,57,60,64,76,79,81,84] |
| **CMOC9 Lack of improved health outcomes and influence on participation**  A visible lack of improvement in health outcomes **(context)** of people from BAFDC means that when people from BAFDC are asked to take part in health and care research studies **(mechanism)** they are unlikely to take part **(outcome).** When asked repeatedly to take part, this leads to feelings of fatigue **(response)** because individuals from BAFDC do not see their participation improving health outcomes within their communities.  This also results in lack of trust **(outcome)** because individuals from BAFDC cannot see the benefits of taking part. | [57-58, 60, 70-71,76] |
| **Implicit and complicit bias** | |
| ***Institutional norms uphold complicit bias (IPT5)*** | |
| **CMOC10 Productive ignorance and complicit bias**  The social exclusion of people from BAFDC in health and care research occurs due to institutional and invisible norms **(context)** that uphold complicit bias, preventing existing research practices and policies, that are perpetuating exclusion, from changing. There is a deficit in the existing system that lacks acknowledgement of structural racism **(context).**This deficit exists partly due to productive ignorance **(mechanism)** . Productive ignorance fails to acknowledge, understand and learn about structural racism. Productive ignorance focusses on getting the job done e.g. reaching recruitment targets and researchers do not feel that they can discuss practices that may be structurally racist, or may even be discouraged from questioning existing practices that may expose structural racism because it feels uncomfortable **(mechanism).** This results in a silencing of conversations **(outcome1)** about structural racism and blame is subsequently shifted through an imbalance of power **(mechanism)** onto Black people. This results in the development of interventions as a way of deflecting responsibility and may seem easier to solve by placing responsibility on the individual to come up with solutions **(outcome 2)** . | [61,74] |
| **CMOC11 The influence of scientific racism and other ideologies**  Ideologies **(contextual influence)** that stem from scientific racism, a belief that there are biological differences between people of different races, may influence the beliefs **(context)** held by some health and care researchers, though these may be implicitly held biases. Scientific racism is upheld through cultural hegemony **(mechanism)** and reinforces hierarchies between racial groups. Cultural hegemony is understood to be an unequal power dynamic between cultures whereby one culture maintains control over norms, values and beliefs. Poor communication about health and care research, that is rushed, with no time for building rapport ,combined with beliefs that biologise people from BAFDC, makes people from BAFDC feel dehumanised **(mechanism),** like they are being used as guinea pigs and can enable scientifically racist beliefs to remain unquestioned. As a result, Black people may experience barriers to participation and exclusion due to unequal treatment **(outcome)**. | [61,65,68-69,71,76,78,80,85] |
| ***Absence of critical reflection by researchers (IPT6)*** | |
| **CMOC12 Deep critical reflection and disrupting the status quo**  There is a lack of critical reflection **(context)** by health and care researchers particularly around their positionality, power and privilege. Health and care researchers feel conflicted at times because of a fear of using racialised language **(mechanism)**. This is upheld through a colourblind ideology **(contextual influence)** that denies cultural differences to be acknowledged as well as the social mechanism of Whiteness that prevents the normalisation of different cultures, or cultural practices. This results in complicit bias as the default and stops health and care researchers from feeling comfortable in talking about **(outcome 1)** how study designs and research practices may be excluding individuals from BAFDC .Critical reflection based upon an emancipatory and social justice praxis could help to disrupt the status quo through action, instead of complicity **(outcome 2).** | [46,59,61,65,67] |
| **Processes that affect inclusion and participation** | |
| ***Relevance of health and care research to the needs of Black people (IPT 7)*** | |
| **CMOC13 Not seeing the value in PPI**  Patient and public involvement (PPI), co-production, or co-design may not always be valued **(context)** by health and care researchers as an essential aspect of developing a health and care research study, or conducted in a way that includes individuals from ethnic minorities in the development of a research study. Health and care researchers may not view building relationships **(mechanism)** with individuals from BAFDC as scientific and thus they under-value, or are indifferent to PPI, co-production and developing skills around cultural competency/humility and consequently lack knowledge and skills of cultural understanding and co-production to be able to involve ethnic minority communities such as BAFDC in the design of health and care research effectively. This may evoke a feeling of being over-researched **(mechanism),** by individuals from BAFDC as they do not see researchers wanting to work with them, nor investing in building relationships and developing research studies with them **(outcome)**. | [46,57,67,72,85-86] |
| **CMOC14 Historical policies, practices and social exclusion**  Historical and existing government policies and practices **(context)**, as well as historical abuses in health and care research, have negatively impacted upon people from BAFDC in the UK, (such as the Windrush Scandal). They have contributed to social exclusion which may impact on inclusion and participation in health and care research. A continuous narrative by government officials through policies that perpetuate structural racism, creates feelings of rejection, instability and a lack of belonging by Black people **(mechanism).**This leads to a lack of trust **(outcome 1),** which is a prerequisite to participation due to wider experiences of racism and racial discrimination continuing to be a major barrier that erodes trust and therefore results in a lack of participation and representation in health and care research **(outcome 2).** | [45-52,59,65,74,76,84,85] |
| **CMOC15 Cultivating openness to health and care research**  Expectations to increase participation **(context)** in health and care research studies by funders, institutions, health and care researchers and health professionals creates pressure to recruit, but a focus on creating an environment that places importance on “openness” to have more general discussions about health and care research **(mechanism),** may reduce this pressure and create better engagement. Shifting the focus to learn more about the beliefs and experiences of individuals from BAFDC and open discussions about health and care research may lead to individuals from BAFDC feeling listened to **(mechanism)** and support the development of more trusting relationships with health and care researchers and healthcare professionals **(outcome).** | [51,61] |
| **CMOC16 Narratives that influence the research design process**  Narratives and scientific knowledge that exist in the health and care research system contribute to upholding deficit thinking **(context),** or ideas about people from BAFDC that silences and misrepresents their lived experience and histories **(mechanism).** Individuals from BAFDC may use storytelling, or counter-storytelling (**mechanism)** exposing oppressive practices and norms which can counteract deficit narratives and ideas about people from BAFDC and thus builds trust **(outcome 1)** and helps to achieve social justice **(outcome 2).** | [48-49,54,69,72,83,85] |
| ***The impact of language (IPT8)*** | |
| **CMOC17 Misalignment of organisational norms and communication of information**  The organisational norms within health and care research system does not align with the needs of people from BAFDC **(context),** creating a lack of appreciation for cultural relevance and competence which influences the way in which information is provided and communicated to people from BAFDC. In addition to the relationship **(mechanism)** individuals have with a health and care researcher (which is the most important for building trust with BAFDC) the accessibility of study information **(mechanism)** is also important but can create a feeling of invasiveness and concern for safety **(mechanism)** if it is not communicated sensitively. A lack of culturally appropriate and sensitive information about studies can negatively influence individuals from BAFDC to decide not to take part in health and care research because information has not been tailored and written for, or with, them **(outcome 1)**. This may also create an unequal power dynamic because of ineffective communication **(outcome 2)**. | [49,52-53,57,65,67,73,84,86] |
